# How informative is the mouse for human gut microbiota research?

**DOI:** 10.1242/dmm.017400

**Published:** 2015-01

**Authors:** Thi Loan Anh Nguyen, Sara Vieira-Silva, Adrian Liston, Jeroen Raes

**Affiliations:** 1 KU Leuven, Department of Microbiology and Immunology, Rega Institute, Herestraat 49, B-3000 Leuven, Belgium.; 2 VIB, Center for the Biology of Disease, Herestraat 49, B-3000 Leuven, Belgium.; 3 Microbiology Unit, Faculty of Sciences and Bioengineering Sciences, Vrije Universiteit Brussel, Pleinlaan 2, B-1050 Brussels, Belgium.

**Keywords:** Gut microbiota, Humanized mouse models, Mouse core gut microbiota, Mouse models, Mouse pan-gut microbiota

## Abstract

The microbiota of the human gut is gaining broad attention owing to its association with a wide range of diseases, ranging from metabolic disorders (e.g. obesity and type 2 diabetes) to autoimmune diseases (such as inflammatory bowel disease and type 1 diabetes), cancer and even neurodevelopmental disorders (e.g. autism). Having been increasingly used in biomedical research, mice have become the model of choice for most studies in this emerging field. Mouse models allow perturbations in gut microbiota to be studied in a controlled experimental setup, and thus help in assessing causality of the complex host-microbiota interactions and in developing mechanistic hypotheses. However, pitfalls should be considered when translating gut microbiome research results from mouse models to humans. In this Special Article, we discuss the intrinsic similarities and differences that exist between the two systems, and compare the human and murine core gut microbiota based on a meta-analysis of currently available datasets. Finally, we discuss the external factors that influence the capability of mouse models to recapitulate the gut microbiota shifts associated with human diseases, and investigate which alternative model systems exist for gut microbiota research.

## Introduction

Murine models have been widely used in biomedical research. Extensive similarities in anatomy, physiology and genetics have allowed numerous inferences about human biology to be drawn from murine experimentation. The advanced knowledge of mouse genetics and the availability of numerous genetically modified mouse models greatly facilitate functional studies. Moreover, their low maintenance cost (as compared with other mammalian experimental models), high reproductive rates and short life cycle are substantial advantages of the mouse model.

In gut microbiota research, mouse models are being increasingly used to study the role and functioning of the gut microbiota and its association with diseases. Alterations in gut microbiota composition and function have been associated with many human pathologies, ranging from metabolic disorders, such as obesity (Le Chatelier et al., 2013; Ley et al., 2006) and type 2 diabetes (Qin et al., 2012), to complex diseases, such as inflammatory bowel disease (IBD) (Manichanh et al., 2012), and autoimmune diseases, such as rheumatoid arthritis (Vaahtovuo et al., 2008) and allergy (Russell et al., 2012). More recently, bidirectional interactions of the gut microbiota on host brain function, through neurohumoral communication (dubbed the gut-brain axis) have also been gaining attention in gastrointestinal disorders, such as irritable bowel syndrome, as well as in more unexpected pathologies, such as autism (De Angelis et al., 2013; Kang et al., 2013; Wang et al., 2013).

Experimental manipulations of murine models in gut microbiota research allow functional and mechanistic research on host-microbe interactions, thus helping to assess causality in disease-associated alterations in gut microbiota composition. Manipulations that are essential to gut microbiota research include host genetic background manipulation (gene knockouts), gut microbiota composition manipulation (controlled inoculation in germ-free or gnotobiotic mice, i.e. germ-free mice administered with external microbes) and ecosystem interventions including dietary interventions, antibiotic treatment and fecal transplantations. Using mouse models in gut microbiota studies has brought more insights into the pathological mechanisms of several diseases, such as defining the role of gut microbiota in the pathogenesis of IBD or in controlling energy balance of the host in obesity. For example, in obesity studies, genetically modified models (such as the *ob/ob* leptin-deficient mouse) and germ-free mouse models are indispensable because they allow interventions that cannot be performed in humans to provide evidence of how gut bacteria influences host metabolism (Bäckhed et al., 2007). Although results from such experiments have yielded important breakthroughs in understanding the dynamic and complex relationship between the gut microbiota and its host, translating such results from murine models to humans remains nontrivial due to the existence of some key differences between the two systems that need to be taken into account. In this Special Article, we compare both model systems with regard to intestinal anatomy and function, and conduct a comparative analysis of the healthy gut microbiota composition in humans and mice, using publicly available fecal microbiota datasets. By investigating these intrinsic differences and external factors shaping the composition of the gut microbiota, we assess the strengths as well as the pitfalls of murine model usage in translational gut microbiota research.

## The anatomy of the mouse and human intestinal tract

Mouse and human are quite similar in physiology and anatomical structures, and this is one of the reasons why mouse models have been widely used in biomedical studies. Particularly, the gastrointestinal tracts in both species are composed of organs that are anatomically similar. However, the anatomy of the mouse and human intestinal tract also have prominent differences (Table 1), which might be shaped by their diverging diets, feeding patterns, body sizes and metabolic requirements.

**Table 1.**
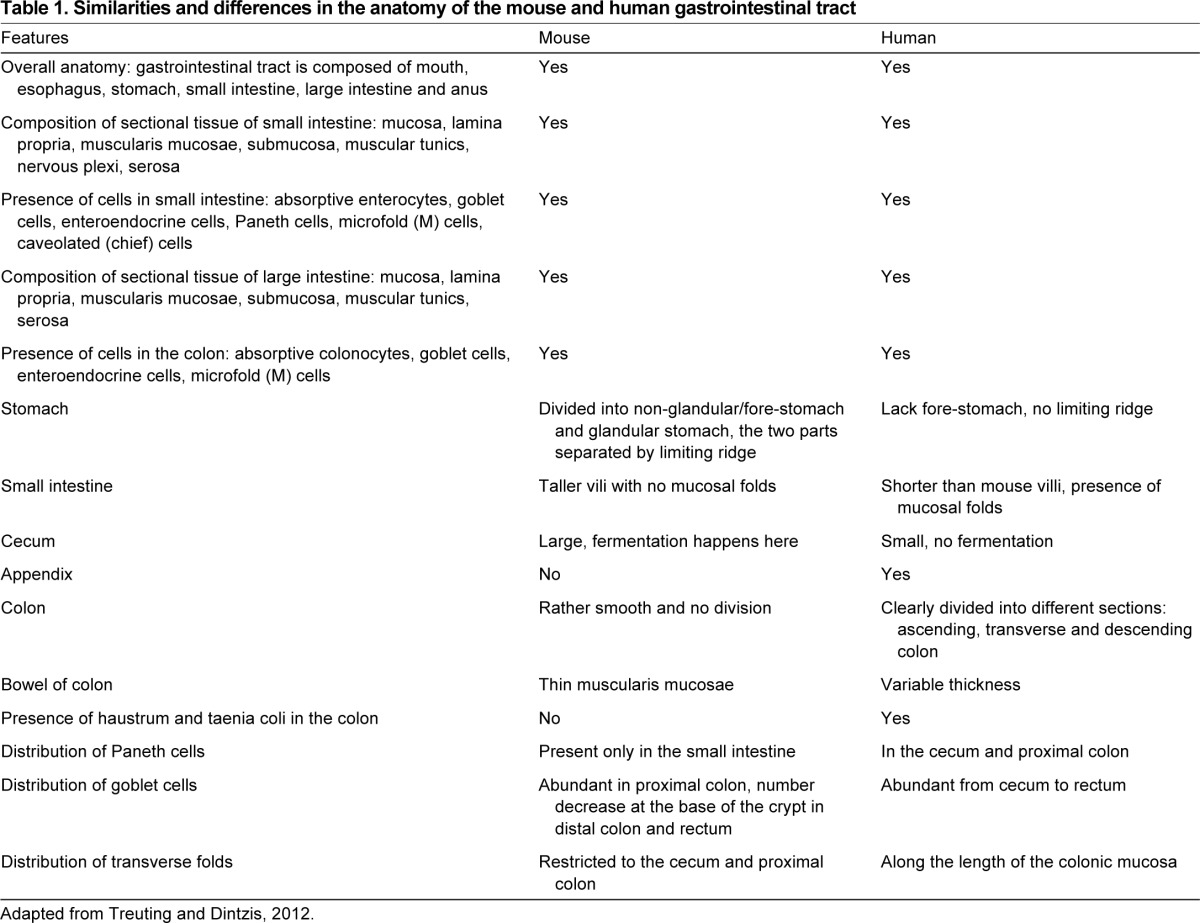
Similarities and differences in the anatomy of the mouse and human gastrointestinal tract

**Fig. 1.**
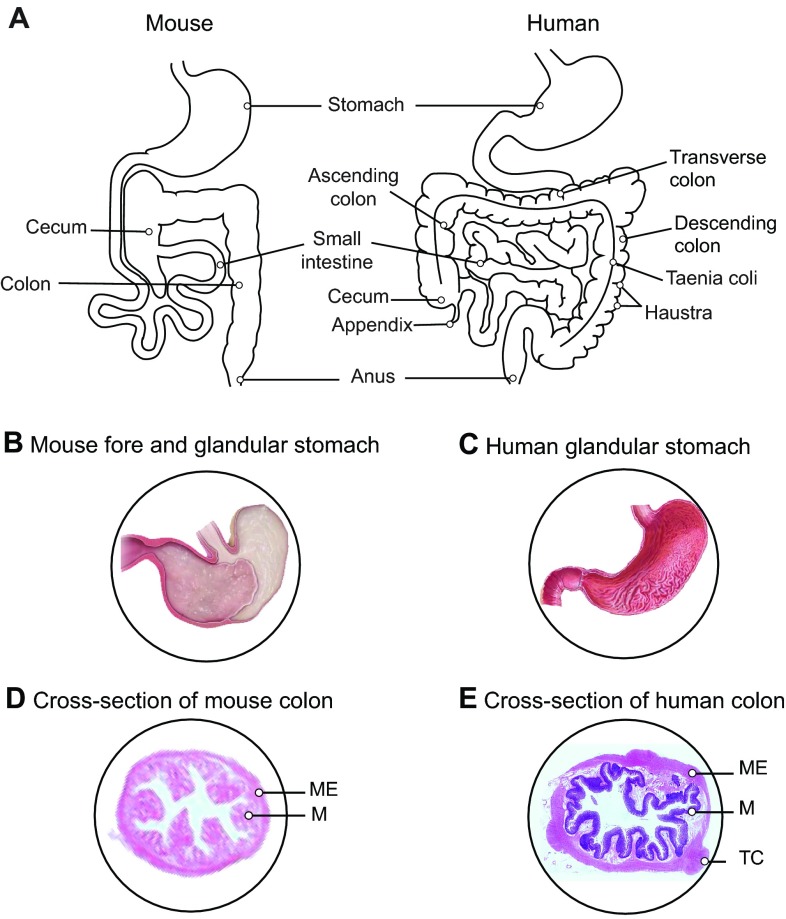
**Gross anatomy of the human and the mouse gastrointestinal tract.** (A) The human colon is divided into different sections (i.e. ascending, transverse and descending colon) with the presence of taenia coli and compartmentalization in haustra, which are absent in the mouse colon. The human stomach is lined with a glandular mucosa (C) that secretes gastric acid, whereas the mouse stomach is divided in two regions – a glandular stomach and a non-glandular or fore-stomach (B). The mouse glandular stomach is responsible for secreting gastric acid, whereas the non-glandular stomach functions as a temporary site of food storage and digestion. (E) Cross-section of a human colon, which has a thicker muscular wall and mucosa compared with the mouse colon (D). M, mucosa; ME, muscularis externa; TC, taenia coli. Panels B and C are reproduced from “Comparative anatomy and histology: A mouse and human atlas” by Piper M. Treuting and Suzy Dintzis, 2012, with permission from Elsevier. D is reproduced from the website http://theses.ulaval.ca/archimede/fichiers/24866/ch07.html with the author’s permission. E is re-used from www.anatomyatlases.org with the author’s permission.

Even though the average ratio of intestinal surface area:body surface area is similar between mice and humans (Casteleyn et al., 2010), this ratio differs greatly between the two species over different sections of the gut. For example, the average small-intestine:colon length ratio is 2.5 in mice versus 7 in humans (Treuting and Dintzis, 2012), and the surface ratio of small intestine:colon is only 18 in mice compared to 400 in humans (Casteleyn et al., 2010). The mouse cecum is also large relative to its total gastrointestinal (GI) tract and is an important site for the fermentation of plant materials as well as for the production of vitamin K and B, which mice reabsorb through coprophagy (Fig. 1). By contrast, the human cecum is relatively small, with an anatomical structure similar to that of the colon and does not hold a clear function (Treuting and Dintzis, 2012). These morphological differences reflect murine adaptation toward an expanded colon and cecum capacity, allowing them to extract nutrients from the relatively larger proportion of indigestible food components in their diet, as compared with humans. Humans also have an appendix, which is absent in mice. The appendix is a vermiform organ attached to the cecum (Fig. 1) and was hypothesized to be a remnant of the cecum under selective pressure of diet. However, the organ has recently been shown to have evolved under multiple environmental factors beyond diet (Smith et al., 2013b). The appendix has also been suggested to act as a repository for beneficial bacteria to replenish the gut microbiota after disturbances (Smith et al., 2013b).

**Fig. 2.**
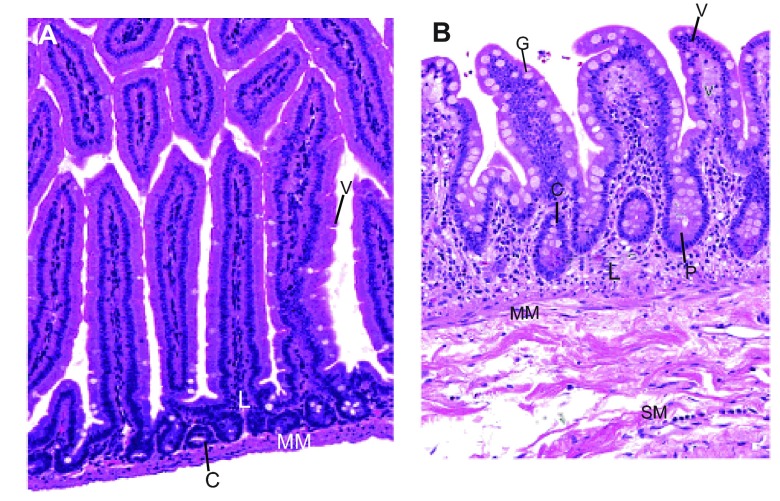
**Anatomical structures of the intestinal wall in mice and in humans.** A and B are taken at the same magnification (20×) and show a section of the small intestinal wall in mice (A) and humans (B), illustrating that mouse intestinal villi (A) are taller than those in humans (B). C, intestinal crypts; G, goblet cells; L, lamina propria; MM, muscularis mucosae; P, Paneth cells; SM, submucosa; V, villi. The images are reproduced from “Comparative anatomy and histology: A mouse and human atlas” by Piper M. Treuting and Suzy Dintzis, 2012, with permission from Elsevier.

Mouse intestinal villi are taller than those of human (Fig. 2). This morphological difference increases the surface area of the mouse small intestine and has been suggested as a compensation mechanism for the lack of mucosal folds in the mouse intestine. The mouse colon is rather smooth with no division, whereas the human large intestine is sub-compartmentalized into pouches (called haustra; see Box 1 for a glossary of terms, and see Fig. 1), which are absent in the mouse colon. In humans, fermentation of dietary carbohydrates is bound to the large intestine, and is not observed in either the vestigial cecum or appendix. These major anatomical differences in the gut compartmentalization between mice and humans, especially with regards to the greater fermentation capacity of mice (in the cecum), probably impact the diversity and composition of the gut microbial communities in the colon. These communities are not only responsible for the fermentation of indigestible food components, but also for the production of essential complements to the host such as vitamin K and B and short-chain fatty acids (SCFAs).

**Box 1. Glossary of scientific terms**

**Adiponectin:** a protein involved in regulating glucose level and fatty-acid breakdown.

**Bacteroidetes:** Gram-negative, non-spore-forming anaerobic bacteria; one of the major bacterial phyla in human and mouse gut microbiota.

**Coprophagy:** consumption of feces.

**Dysbiosis:** imbalance of human-associated microbial communities, usually related to disease, with compositional (microbial species) and functional (microbial metabolism) deviation from the normal microbiota.

**Enterotype:** classification of humans (or other host organisms) based on the composition of their gut microbiota.

**Firmicutes:** Gram-positive, endospore-producing anaerobic bacteria; one of the major bacterial phyla in human and mouse gut microbiota.

**Gut-brain axis:** bidirectional signaling between the gut microbiota and the host brain, affecting normal homeostasis and contributing to disease.

**Haptens:** small molecules that can elicit immune responses when attached to larger non-immunogenic carriers.

**Haustra:** small pouches segmenting the large intestine, caused by tension of shorter exterior muscle ribbons (cf. taenia coli).

**Humanized gnotobiotic mice:** mice born germ-free and inoculated with a human gut microbiota sample.

**Isobiotic mice:** genetically identical mice, only differing in microbiota composition.

**Metagenomic analysis:** study of a microbial community by sequencing the genetic material pool from an environmental or clinical sample.

**Muscularis mucosae:** thin muscle layer, separating the lamina propria mucosae from the submucosae.

**Taenia coli:** three longitudinal smooth muscle ribbons along the outside of the colon.

In addition to the macroscopic differences, the microscopic structure of the intestinal tract of mice and humans also differ (Treuting and Dintzis, 2012). The mouse colon is composed of thin muscularis mucosae (see Box 1 and Table 1) with no discernible sub-mucosa, whereas the human colon is covered by a thicker mucosal wall. Another difference is the presence of transverse folds along the length of the colonic mucosa in humans, whereas these folds are restricted to the cecum and proximal colon in mice. These differences in colonic micro-compartmentalization and structuring might contribute to the creation of diverse ecological micro-niches hosting differing microbial communities.

At the cellular level, there are also a number of notable differences between humans and mice. The first example is the distribution of mucin-producing goblet cells (Table 1). In mice, these cells are abundant along the surface of intestinal crypts in the proximal colon but, in the distal colon and rectum, their number decreases at the base of the crypt. Conversely, in humans, goblet cells are abundant from cecum to rectum. The second example is the difference in distribution between mice and humans of another type of intestinal epithelial cell – the Paneth cell (Table 1). Paneth cells secrete antimicrobial compounds into the lumen of the small intestine. They are rare but present in the cecum and proximal colon of humans, whereas, in the mouse, these cells are entirely absent in the colonic mucosa and uniquely found in the cecum. In addition to location differences, the amount of defensins (peptides involved in the host defense) secreted by Paneth cells, their storage and secretion were also reported to be different between mice and humans (Cunliffe et al., 2001; Ghosh et al., 2002; Ouellette and Selsted, 1996). These differences in distribution of both goblet cells and Paneth cells between the two organisms suggest differences in local immune responses, which might shape the composition of the gut microbiota.

Overall, the mammalian digestive tract is strongly conserved, with major differences between species being likely driven by diet. Given their shared omnivorous nature, humans and mice thus share strong similarities. However, humans have evolved towards a smaller cecum and colon and relatively longer small intestine as compared to the murine system. In mice, fermentation of indigestible food components is compartmentalized in the cecum, whereas, in humans, fermentation takes place in the colon, and the cecum is vestigial (Fig. 1). The morphology of mouse and human colons also differs: the human colon is divided into haustra, whereas the mouse colon is rather smooth. Cells that are essential to intestinal integrity and host-microbiota equilibrium, such as goblet and Paneth cells, are also conserved between the two species, although there are differences in distribution. Although these differences do not mean that the murine model is not valuable to study host-microbiota interactions, care must be taken in making direct parallels between murine and human gut with regard to microbiota composition, because host-microbiota co-evolution could have been influenced by these anatomical divergences.

## Human and mouse gut microbiota composition in health and disease

Given the considerations mentioned above, in this section we further analyze the similarities and discrepancies between the murine and human gut microbiota composition, as well as their respective responses upon dietary interventions. Finally, we compare disease-associated microbiome shifts between the two organisms.

### Composition of the gut microbiota in healthy humans and mice

**Fig. 3.**
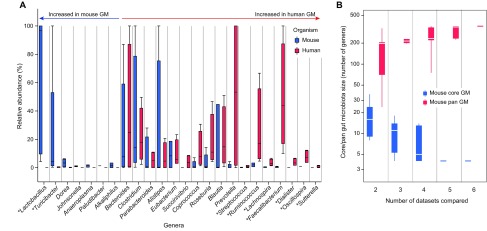
**Meta-analysis of mouse and human fecal microbiota based on published 16S rDNA sequencing data.** (A) Comparison of human and mouse healthy adult gut microbiota. The relative abundances of genera in the gut microbiota of both organisms [four human datasets (Human Microbiome Project Consortium, 2012; Yatsunenko et al., 2012) and five mouse datasets (Nagy-Szakal et al., 2012; Riboulet-Bisson et al., 2012; Ubeda et al., 2013; Ward et al., 2012; Zenewicz et al., 2013)] are ordered according to their overrepresentation in either mouse or human gut microbiota (non-parametric Wilcoxon Z score). Genera with significant differences (*P*<0.05) between human and mouse gut microbiota are annotated with an asterisk. Note that none of these differences are significant after multiple testing corrections. Note that only five mouse datasets are used in this comparison because the dataset from Cho et al. (Cho et al., 2012) does not include data for abundance and thus cannot be used for comparison of relative abundances between mouse and human gut microbiota. (B) Mouse core and pan-gut microbiota size in all possible combinations of the six mouse gut microbiota datasets (Cho et al., 2012; Nagy-Szakal et al., 2012; Riboulet-Bisson et al., 2012; Ubeda et al., 2013; Ward et al., 2012; Zenewicz et al., 2013). The pan-gut microbiota is the set of genera found at least once in any of the datasets compared (union set), whereas the core gut microbiota is the set of genera found in all compared datasets (intersection set). It should be noted that Zenewicz’s dataset overlaps poorly with the others.

Overall, the gut microbiota of human and mice are dominated by two major phyla, Bacteroidetes and Firmicutes (Eckburg et al., 2005; Ley et al., 2005; Ley et al., 2006). However, when exploring deeper taxonomic classifications, Ley et al. showed that 85% of bacterial genera found in the mouse gut microbiota are not present in human (Ley et al., 2005). Several differences in research techniques hamper the comparison of the murine and human microbiome. First, most human gut microbiome studies use stool samples, whereas cecal contents are usually used in mouse gut microbiome studies (with the exception of longitudinal studies, where pellets are sampled). Furthermore, the composition of the human gut microbiota has been investigated in several studies (Arumugam et al., 2011; Human Microbiome Project Consortium, 2012; Qin et al., 2010; Yatsunenko et al., 2012) using both metagenomic and 16S rDNA sequencing approaches, whereas the standard choice for mouse studies is mostly 16S rDNA sequencing (Brinkman et al., 2011; Brinkman et al., 2013; Hildebrand et al., 2013; Kellermayer et al., 2011; Riboulet-Bisson et al., 2012; Ubeda et al., 2013; Ward et al., 2012; Zenewicz et al., 2013), although metagenomic analysis has also begun to be used in mouse studies (Wang et al., 2014). Here, we compare the composition of mouse and human gut microbiota based on all currently available 16S rDNA sequenced data from fecal samples of healthy adults (Fig. 3). Considering the restrictions referred to previously, the analysis is based on a limited number of samples: five murine fecal 16S rDNA studies (Nagy-Szakal et al., 2012; Riboulet-Bisson et al., 2012; Ubeda et al., 2013; Ward et al., 2012; Zenewicz et al., 2013) and four public 16S rDNA healthy adult human datasets (Human Microbiome Project Consortium, 2012; Yatsunenko et al., 2012).

In these datasets, we found 79 genera occurring in both human and mouse gut microbiotas. The relative abundances of most of the dominant genera in mouse and human are quite different (Fig. 3A; supplementary material Table S1). Genera with high abundance in human gut microbiota, as compared to mouse gut microbiota, include *Prevotella*, *Faecalibacterium* and *Ruminococcus*, whereas *Lactobacillus*, *Alistipes* and *Turicibacter* are more abundant in mouse gut microbiota (supplementary material Table S1). *Clostridium*, *Bacteroides* and *Blautia*, on the contrary, have a similar relative abundance in both organisms. However, none of the differences found between human and mouse gut microbiota survives multiple testing corrections due to the limited size of the dataset, so these results should be considered as exploratory. Although it is important to keep in mind the major inter-organism variations, as well as technical discrepancies in the gut microbiota studies compared above (such as different human populations, mouse houses, mouse strains, sample handling, processing techniques and method of analysis), the results nonetheless mostly agree with current knowledge. For instance, *Prevotella* abundance is known to be low in mouse gut microbiota (Hildebrand et al., 2013) and *Faecalibacterium* is reported to be one of the dominant members in healthy gut microbiota in humans, as it has been suggested to be the marker for anti-inflammatory gut microbiota in IBD patients in remission (Sokol et al., 2008; Sokol et al., 2009).

We then defined the core gut microbiota of mice, i.e. the taxa that are always present in each individual gut microbiota (intersection set of genera across samples). The comparative analysis of six mouse gut microbiota datasets (Cho et al., 2012; Nagy-Szakal et al., 2012; Riboulet-Bisson et al., 2012; Ubeda et al., 2013; Ward et al., 2012; Zenewicz et al., 2013) (Fig. 3B; supplementary material Table S2) shows that the core mouse gut microbiota plateaus down to four genera [note that this number increases to 13 genera if we exclude the limited Zenewicz’s dataset (Zenewicz et al., 2013), which overlaps very poorly with the other datasets (data not shown)]. In humans, using 16S rDNA cloned sequences from 17 individuals, Tap et al. found seven genera that were common in 50% of the cohort (Tap et al., 2009). On the other hand, a study by the MetaHIT consortium investigating metagenomes from 124 European individuals discovered that 90% of the individuals of the cohort share a common core of nine genera, at a 10% sequence coverage threshold (i.e. species being only considered present if a minimum of 10% of their genome was recovered in the sequenced metagenome) (Qin et al., 2010). More recently, Martínez et al. defined a core set of 24 bacterial genera (Martínez et al., 2013) in a longitudinal study of three healthy human individuals during one year, which would constitute a ‘healthy’ core microbiota set. Thus, first observations indicate that the murine core microbiota could be smaller than the humans’, but this needs further follow up to be sure that this result is not driven by insufficient sequencing depth in these first datasets.

The total set of genera found across the six mouse gut microbiota datasets previously referred to (pan-gut microbiota: union set of genera across samples) cumulates to 352 genera (Fig. 3B), of which only 44 are found in more than three mouse gut microbiota datasets. This number is higher than the one observed in humans: the overall stool microbiota richness (i.e. the number of different genera in the population) was estimated at 226 bacterial genera across 208 donors, using the data from the Human Microbiome Project Consortium (Li et al., 2013).

Recently, a study by Krych et al. identified about 80 genera that are shared between mice and humans (with abundance threshold set to 0.19% to filter out variations due to sequencing method or sequencing depth) (Krych et al., 2013), a number that is very similar to the 79 found in our meta-analysis. The authors proposed that multiple genera are present exclusively in the human gut microbiota: *Faecalibacterium*, *Mitsuokellla*, *Megasphera*, *Dialister*, *Asteroleplasma*, *Succinivibio*, *Sutterella*, *Paraprevotella* and *Phascolarctobacterium*, with *Mucispirillum* being the only genus exclusive to mice (Krych et al., 2013). However, as a case in point, *Faecalibacterium* was found in the mouse gut microbiota datasets from Ward (Ward et al., 2012), Riboulet-Bisson (Riboulet-Bisson et al., 2012) and Nagy-Szakal (Nagy-Szakal et al., 2012). The genus was also detected in a mouse study from Werner et al. (Werner et al., 2011). These examples indicate the difficulty of inferring a bacteria’s exclusivity to an organism based on a lack of its observation in community studies, where only a fraction, albeit an increasing one, of bacterial genera are accessible with current techniques. Such conclusions are thus affected by sequencing depth, the age of the mice/human subjects, the mouse strains/human populations chosen, the different microbiota pools in different laboratories and by other practical factors involved in gut microbiota studies. In addition, the effect of multi-generation specific pathogen free (SPF) conditions might serve to reduce the murine gut microbiota diversity to below that of healthy wild mice. Accordingly, a study by Linnenbrink et al. reveals that wild mice have a greater bacterial diversity in their cecal microbiota than laboratory mice housed in SPF facility (Linnenbrink et al., 2013). Another important point is that, in most current analyses, low-abundance genera on the boundary of sequencing depth are overlooked. However, a recent study investigating microbiota across human body habitats suggests that low-abundance genera might play more important roles, because they are ubiquitous in different sites of the body (Li et al., 2013). Together, these findings highlight that inter-study variations should be considered more carefully, and that a final conclusion on discrepancies and similarities between humans and mice has not been reached.

One important finding in the inter-individual diversity in human gut microbiota was the observation of the existence of a limited set of possible gut communities – termed enterotypes (Arumugam et al., 2011). Although the degree of distinctiveness between these clusters of human gut microbiota is still a matter of debate (Ding and Schloss, 2014; Koren et al., 2013), a consensus on their usefulness as a stratification tool is growing (Moeller and Ochman, 2014). Enterotypes were also identified in the laboratory mouse gut microbiota (Hildebrand et al., 2013), being dominated by Lachnospiraceae and Ruminococcaceae or Bacteroidaceae and Enterobacteriaceae, respectively. Although the cause of stratification of human and mouse individuals into enterotypes is still unknown, there is a noticeable parallel between dominant bacterial families of the mouse and human enterotypes. Namely, one mouse enterotype is dominated by Lachnospiraceae/Ruminococcaceae, similarly to the human Ruminococcaceae enterotype (also known as enterotype 3). In addition, the second mouse enterotype, dominated by Bacteroidaceae/Enterobacteriaceae, is similar to the human *Bacteroides* enterotype (enterotype 1) (Arumugam et al., 2011). Interestingly, two enterotypes were also identified in wild mice, dominated by *Bacteroides* and *Robinsoniella*, respectively (Wang et al., 2014). Moreover, the laboratory mouse enterotypes were found to correlate with species-richness and inflammation: mice belonging to the low species-richness enterotype (Bacteroidaceae/Enterobacteriaceae) had higher levels of calprotectin, a marker of inflammation. This result is consistent with what has been recently found in studies of human obesity (Cotillard et al., 2013; Le Chatelier et al., 2013), in which low species-richness individuals were found to have more pronounced inflammation, and to be dominated by Bacteroidetes and Proteobacteria, the same bacterial groups that dominated the ‘inflamed’ mouse enterotype.

Overall, these observations show that clear differences can be observed at the level of specific genus/species abundances between the murine and human gut microbiota. The observed differences might be caused by intrinsic differences between these two mammalian systems, but also by various confounding factors ranging from diet to exposure to pathogens. At the same time, overall community composition rules as well as the factors driving them might be similar (e.g. enterotypes). Thus, although absolute comparisons might be difficult, murine models are likely relevant for studying the processes responsible for microbiota variation and shifts upon disturbance.

### Do gut microbiota shifts in murine models mimic those reported in human disease?

Mouse models are a powerful tool to study the underlying mechanisms of gut-microbiota-associated diseases. Given the anatomical and compositional differences in the healthy control individuals, we review the concordance in the major shifts in gut microbiota associated with the most popular gut microbiota-related diseases: obesity and IBD.

#### Obesity

The influence of diet on the gut microbiota has received increasing research attention over recent years. With the rising incidence of metabolic disturbances, such as obesity and diabetes in Western countries, the impact of the ‘Western’ diet (high in simple carbohydrates and animal fats) on the gut microbiota and our health is a key question in this field. Many studies have been conducted on mice or humanized mouse models (i.e. germ-free mice administered human gut microbiota), which have been fed diets that are high in fat or saturated/unsaturated fat to investigate changes in the gut microbiota (Ley et al., 2006; Liu et al., 2012; Turnbaugh et al., 2009b; Wu et al., 2011; Zhang et al., 2012). Some common trends have emerged from this research. For instance, mice fed on a high animal-fat diet show a decreased Bacteroidetes:Firmicutes ratio in their gut microbiota (Murphy et al., 2010; Zhang et al., 2012). This shift is driven by more complex alterations at lower taxonomic levels within the phylum Bacteroidetes: most genera, including *Prevotella* and *Roseburia*, decreased in abundance (Neyrinck et al., 2011), whereas other genera of this phylum, such as *Barnesiella*, *Bacteroides* and *Alistipes*, significantly increased (Zhang et al., 2012). Interestingly, the overall trend observed in mouse studies agrees with that found in human studies. Human dietary studies have revealed that the *Bacteroides* enterotype in humans is associated with people whose diet contains more animal fats over long periods of time, whereas the *Prevotella* enterotype is found to predominate in people consuming more carbohydrates (Wu et al., 2011). A study comparing European and rural African children also confirmed this link between microbiota and diet (De Filippo et al., 2010). African children, who eat fiber-rich diets, had a higher abundance of specific Bacteroidetes (*Prevotella* and *Xylanibacter*), a reduced amount of Firmicutes and decreased amounts of Proteobacteria (*Shigella*, *Escherichia*), compared with European children. The study suggested that European children with a high-fat diet harbor the *Bacteroides* enterotype, which might predispose them to many metabolic diseases (Le Chatelier et al., 2013). Overall, this highlights that the use of diet intervention in murine models, including the use of humanized gnotobiotic mice, can mimic the changes in gut microbiota that occur in relation to human diet.

However, the dynamics of enterotypes during diet interventions do show discrepancies between human and mouse studies. Wu et al. followed enterotype switches in participants with controlled diets (randomized to a high-fat/low-fiber or low-fat/high-fiber diets) over a period of 10 days (Wu et al., 2011). There was no switch between *Bacteroides* enterotype, which is associated with animal protein, and *Prevotella* enterotype, which links to diets with more carbohydrates and simple sugars. The authors therefore suggested that a long-term dietary intervention might be needed to modify an individual’s enterotype (Wu et al., 2011). On the other hand, a study on wild mice by Wang et al. showed that feeding wild mice with chow diet can change mice enterotypes as quickly as within 1 week, from the *Bacteroides* enterotype (associated with protein metabolism) to the *Robinsoniella* enterotype (associated with carbohydrate metabolism) (Wang et al., 2014). The discrepancies in these results suggest that humans need more time for the diet interventions to change their enterotypes given the more drastic changes in diet in the wild mouse study. The discrepancies might otherwise illustrate the intrinsic differences, e.g. the effect of genetic background on enterotype identities, as well as external confounding factors, e.g. environmental influences, which affect the two organisms.

Similarly, there is controversy over the gut microbiota changes observed in human and mouse obesity studies. For instance, in some human studies, obese individuals have been reported to have an increased Firmicutes:Bacteroidetes ratio (Ley et al., 2006; Turnbaugh et al., 2009a), which decreases when submitted to a low-calorie diet (Ley et al., 2006). In mouse studies, besides feeding wild-type mice *ad libitum* with a high-fat diet, leptin-deficient (*ob/ob*) mice have also been used as a model for obesity. These mice, which lack the gene encoding the hormone leptin, which has a crucial role in the regulation of the appetite, have increased food intake as compared with wild-type mice and ultimately become obese. The gut microbiota of this obese mouse model was also found to have an increased ratio of Firmicutes:Bacteroidetes (Ley et al., 2005; Murphy et al., 2010), as seen in some human studies mentioned above (Ley et al., 2006; Turnbaugh et al., 2009a). Moreover, a genome-wide association study of obesity in mice identified genes associated with obesity in mice that overlap with some genes involved in human obesity (Parks et al., 2013). This genetic background overlap suggests conserved mechanisms for obesity susceptibility across mammalian species. Conversely, other studies reported conflicting results for the ratio of Firmicutes to Bacteroidetes, in which overweight and obese individuals were found to have reduced Firmicutes and increased Bacteroidetes (Schwiertz et al., 2010) or have no change in proportions of either Bacteroidetes or Firmicutes (Duncan et al., 2008). A reduced ratio of Firmicutes:Bacteroidetes was also found in a study linking gut microbiota composition and metabolic adaptations in mice on high-fat diets (Serino et al., 2012). The discrepancies in the importance of the Firmicutes:Bacteroidetes ratio found across studies could be linked to the heterogeneity in age of subjects, because the ratio of Firmicutes:Bacteroidetes has been found to change with age (Mariat et al., 2009), or to the different sample-processing/analyzing techniques used in these studies. Another possibility is that focusing on the ratio of Firmicutes:Bacteroidetes, which does not completely capture the compositional changes of the gut microbiota associated with obesity, is an overly simplistic metric to study metabolic disorders.

The mechanisms by which gut microbiota contribute to the pathophysiology of obesity have been investigated in many mouse studies and, thanks to their results, we can now draw a clearer picture of the impact of gut microbiota on maintaining energy balance of the host. Using wild-type and leptin-deficient (*ob/ob*) mice, Turnbaugh and colleagues proposed that the obese gut microbiome has an increased capacity to harvest energy from the host diet (Turnbaugh et al., 2006). The obese gut microbiome was shown to produce a higher level of monosaccharides and SCFAs, which supply the host with extra energy from indigestible food components, as compared with lean animals. The higher concentration of SCFAs in feces of obese compared with lean individuals was also observed in humans (Schwiertz et al., 2010). In addition, by using germ-free and leptin-deficient (*ob/ob*) mice, Bäckhed and colleagues were able to suggest other mechanisms by which the gut microbiota could regulate fat storage in the host (Bäckhed et al., 2007). Particularly, the gut microbiota can control fatty-acid oxidation in the host via suppression of the AMP-activated protein kinases (AMPKs). By contrast, the gut microbiota can also induce fat storage in the host by suppression of fasting-induced adipose factor (Fiaf) (Bäckhed et al., 2007). These results are valuable in understanding the pathology of obesity because they determine the inside mechanisms of how gut microbiota can contribute to energy balance of the host. Although it remains to be elucidated whether the same mechanisms exist in humans, this example well illustrates the usefulness of mouse studies in performing experiments that cannot be done in humans.

#### Inflammatory bowel disease

The development of IBD has been linked to genetic factors, infectious pathogens and alterations in the composition of the gut microbiota (Jostins et al., 2012; Khor et al., 2011), even though the respective causal importance of these factors still awaits clarification. With respect to the role of the gut microbiota, large inter-individual variations among IBD patients regarding genetic background, stage of the disease and diet have made it difficult to identify clear associations between compositional changes in the gut microbiota and the pathology. The comparison of trends of alterations in gut microbiota composition in IBD between human and model organisms is complicated by the fact that IBD encompasses a heterogenous group of diseases, including Crohn’s disease (ileal, colon), ulcerative colitis and intermediate colitis, all of which can also be at different stages (for example, active or in remission). This said, some prominent changes in the gut microbiota have been concomitantly observed in independent IBD studies using a variety of techniques on different cohorts, such as reduced bacterial diversity in both Crohn’s disease and ulcerative colitis patients (Andoh et al., 2012; Nemoto et al., 2012; Sokol et al., 2006; Walker et al., 2011), a reduction in the levels of the anti-inflammatory bacterium *Faecalibacterium* (Andoh et al., 2012; Joossens et al., 2011; Sokol et al., 2009; Willing et al., 2009), as well as of *Clostridium coccoides* and *Bifidobacterium* (Sokol et al., 2009), and an increase in abundance of Enterobacteriaceae (such as *E. coli*) (Duboc et al., 2013; Joossens et al., 2011; Willing et al., 2009; Willing et al., 2010).

**Fig. 4.**
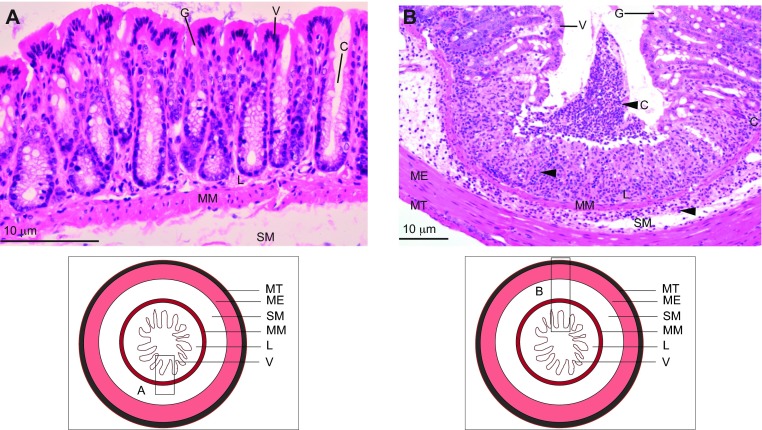
**Histological features of murine DSS-induced colitis.** (A,B) Histology images of colon cross-sections from control (A) and dextran sulfate sodium (DSS)-treated (B) mice. The diagram under each panel illustrates an outline of a mouse colon cross-section with boxes A and B indicating the histological regions shown in panels A and B, respectively. *Mir29*-knockout mice from a Black6 background were treated either with water (control animals) or with 1.5% DSS for 8 days prior to examination. Whereas the cross-section of the colon wall from a control mouse shows normal morphology (A), the colon of the DSS-treated mouse (B) shows alterations in the mucosal epithelium such as cell necrosis, and loss of structure of both villi and intestinal crypts (C; indicated by arrowhead). In addition, infiltration of immune cells such as macrophages, neutrophils, eosinophils and lymphocytes (indicated with arrowheads) is found in the colonic lamina propria (L) and submucosa (SM), indicative of the inflammatory status of the DSS-treated mouse. C, intestinal crypts; G, goblet cells; L, lamina propria; ME, muscularis externa; MM, muscularis mucosae; MT: muscularis tunics; SM: submucosa; V: villi.

Increasing numbers of mouse models have been created and used in IBD research, with the aim of mimicking the disease’s pathophysiology in humans. In general, these models were created by introducing genetic modifications in mice that resemble the genetic defects of IBD patients, or by relying on external disturbances to induce disease. The latter include biological agents, such as infective pathogens (for example, *Helicobacter hepaticus* and *Citrobacter rodentium*), or chemicals, such as dextran sulfate sodium (DSS) and 2,4,6-trinitrobenzene sulfonic acid (TNBS), which cause the initial damage that leads to chronic intestinal inflammation (Peloquin and Nguyen, 2013; Wirtz et al., 2007). However, most mouse models of colitis do not fully recapitulate the pathophysiology of human IBD and generally only cause colonic inflammation (Fig. 4), which is more similar to ulcerative colitis (reviewed in Wirtz and Neurath, 2007 and discussed further below). Despite this, the major gut microbiota shifts that have been observed in different colitis mouse models are similar to those found in human IBD studies. In these models, gut bacterial diversity was found to be reduced, with certain shifts in gut microbiota profiles being observed, such as increases in Enterobacteriaceae (*Escherichia*), Bacteroidaceae (*Bacteroides*) and Ruminococcaceae (Berry et al., 2012; Lupp et al., 2007; Wohlgemuth et al., 2009). However, the reduction in the anti-inflammatory bacterium *Faecalibacterium prausnitzii*, which is observed to occur in many human IBD studies, was not observed in murine colitis models. In addition, *Akkermansia* was found to be reduced in abundance in a human ulcerative colitis study (Vigsnæs et al., 2012), but was increased in the DSS-induced mouse model (Berry et al., 2012). Mouse studies have also detected changes in abundance of bacterial phylotypes that solely happen in mice, such as of Tenericutes and Deferribacteriaceae (Nagalingam et al., 2011). Conversely, changes in the diversity of TM7 phylotypes (Kuehbacher et al., 2008) or in the abundance of *Roseburia* (Willing et al., 2010) were found only in human Crohn’s disease studies. Vereecke et al. recently developed a mouse model prone to spontaneous colitis (Vereecke et al., 2014), while studying A20 – an inhibitor of both NF-κB and apoptotic signaling that has previously been associated with susceptibility to IBD in humans (Catrysse et al., 2014; Ma and Malynn, 2012). Deleting A20 in intestinal epithelial and myeloid cells in mice (*A20*^IEC/myel-KO^) induced intestinal epithelial apoptosis, Paneth and goblet cell loss, and gut microbiota dysbiosis. The latter was characterized by a reduced bacterial diversity and altered composition as compared with wild type. Furthermore, the mouse-specific genus *Mucispirillum* was significantly increased in *A20*^IEC/myel-KO^ mice and was thus suggested as a biomarker for spontaneous colitis in this model. Interestingly, mucosal expression levels of A20 in humans have been put forward as a prognosis marker for response to anti-TNF treatment in IBD patients (Vereecke et al., 2014).

Overall, these results show that obesity and IBD have quite different ‘translatability’ from models to humans, and illustrate both the potential and the drawbacks of this process.

## Practical issues influence the composition of human and murine gut microbiota

The murine and human gut microbiota are not only intrinsically different, but are also affected by environmental factors. These factors can be confounders but can also be controlled for with appropriate experimental design in mouse studies. Here, we discuss how the genetic backgrounds of mouse models, as well as environmental factors, contribute to the overall composition of the mouse gut microbiota.

### Housing conditions

To prevent mice in gut microbiota studies from being contaminated by pathogens from the surrounding environment, they are usually housed in clean facilities, such as in SPF conditions, where hygiene is strictly controlled. Although the microbes allowed in SPF facilities are strictly regulated, each mouse house is home to a distinct pool of microorganisms, including a variety of pathogens that come from contaminations or adventitious infections (Jacoby and Lindsey, 1998; Pritchett-Corning et al., 2009; Taylor et al., 2007). Because mice are housed in a semi-closed environment in which the types of microbes and other environmental factors are partly controlled, their microbiota, including their gut microbiota, depend largely on the microbe pool of the mouse house, on the other mice present and possibly even on their carers, who can unintentionally pass their microbes onto housed mice. As such, the gut microbiota of laboratory mice cannot be considered to fully represent that of normal, free-living mice, and adds to the differences with human gut microbiota, which is exposed to a wide range of environmental cues. It is also possible that, in such a semi-closed system, stochastic effects will influence the available microbiota pool over time (McCafferty et al., 2013). Notably, the accidental discovery of segmented filamentous bacteria that account for the differences in microbiota of mice from two different mouse vendors (Ivanov et al., 2009) highlights the variations in gut microbiota composition brought about by different housing conditions.

It is not only external environmental factors that affect mice in mouse houses; ‘in-house’ interactions with cage mates also influence the composition of their gut microbiota. Within one cage, for example, mice will engage in coprophagy in order to obtain additional nutrients from fecal matter (Soave and Brand, 1991). This activity is considered to be the main reason for the gradual homogenization of the gut microbiota between co-housed mice, as shown by multiple studies (Elinav et al., 2011; Zenewicz et al., 2013). Another factor to be taken into account in gut microbiota research is the maternal transmission of the microbiota in mammals at birth, which is reported to be an important confounding factor in mouse studies (Benson et al., 2010; Friswell et al., 2010; Ley et al., 2005). Microbes from the maternal vaginal, fecal and skin microbiota, are the first ones to occupy the gut microbiota. Maternal inoculation was shown to influence the composition of gut microbiota across four generations (Benson et al., 2010), and is thus an important factor shaping the composition of the gut microbiota. However, the importance of the effect of this initial inoculate on the final composition of the adult microbiota is not clear compared with dietary, physiological, host genetic and stochastic effects during the development of the animal (Hildebrand et al., 2013; Spor et al., 2011). Furthermore, in closed housing conditions, where mice are confined in a limited space, stress related to human handling, noise and social contacts with cage mates is considered to affect gut microbiota composition (Ma et al., 2012). Specifically, it has been shown that animals behave differently when they are caged in isolation (Blanchard et al., 1991). Although eliminating stress induced by conspecific aggression, isolation is a source of stress in social animals. Indeed, it has been shown that mice in groups are buffered against stress and that they show more positive signs of health such as faster wound healing and a lower level of stroke-induced neuronal death (DeVries et al., 2007; Galley et al., 2014). This is relevant within the context of increasing evidence that there is bidirectional communication between the nervous system and the gut microbiota. Given the nature of these interactions, stress could alter gut microbiota composition (Bercik et al., 2012; Cryan and O’Mahony, 2011; Galley et al., 2014; Neufeld et al., 2011).

### Dietary impacts

Usually, laboratory mice are fed a standardized chow diet, a closed formula diet in which the exact amount of each ingredient is not disclosed by the producer. The nutritional content of chow varies from batch to batch, depending on agronomical market fluctuations. Even with these variations, chow diet is composed mainly of plant materials and thus differs considerably to the composition and variation in a human daily diet. In most cases, mice being used in an experiment are fed the same diet, obtained from the same supplier, throughout the experiment. Chow diet might differ from human diets in components that have a significant impact on gut microbiota composition. As a case in point, coumestrol, a phytoestrogen, from alfalfa (clover) is used in rodent diets, which increases the ingestion of estrogen equivalents by mice compared with humans (Degen et al., 2002). In fact, changes in gut microbiota composition have been found to follow changes in the estrogen content in diets of both humans and mice (Akaza, 2012; Menon et al., 2013). Some xenobiotics, such as antibiotics, can also strongly affect the gut microbiota composition and function (Maurice et al., 2013). Humans, through diet or treatments, are often exposed to such compounds and their potential synergistic effects. The mouse models have the advantage of allowing researchers to control for the impact of such compounds on the gut microbiota, by either minimizing exposure or recording levels of exposure.

Furthermore, dietary variations between humans represent a large potential source of gut microbiota inter-individual variation, which would not be detected in lab mice under uniform chow diet. For instance, the *Prevotella* enterotype is known to be sensitive to diet changes and is linked to a high carbohydrate/fiber diet in humans (De Filippo et al., 2010; Liu et al., 2012; Willing et al., 2010; Wu et al., 2011). In both mouse (Neyrinck et al., 2011) and human (Fernandez-Raudales et al., 2012; Wu et al., 2011) gut microbiota studies, the abundance of *Prevotella* was found to change upon dietary intervention. Furthermore, Wang et al. have shown that hosting wild mice in a laboratory environment on chow diet causes a shift in enterotypes, from the original *Bacteroides*-dominated (with microbial metabolism more dedicated to protein degradation) to the *Robinsoneilla*-dominated (more dedicated to carbohydrate degradation) enterotype (Wang et al., 2014).

In summary, it is essential to keep in mind that the murine models’ controlled diet might skew analyses by focusing only on a subset of the mouse gut microbiota inter-individual variance. On the other hand, controlling mouse diets provides a clearer experimental setup to disentangle the associations between the gut microbiota composition and function, and the perturbations studied, such as disease and host physiology (Box 2).

**Box 2. Advantages and limitations of mouse models used in gut microbiota research**

**Advantages**

- Allow interventions that are not possible in humans to study the causal role of gut microbiota in health and disease.
- Comprehensive knowledge of mouse genetics and availability of numerous genetically modified mouse models more than any other models.
- Low cost of maintenance, high reproductive rate, short life cycle.
- Omnivorous mammal, with gut physiology and anatomy comparable to the human.
- Allow targeting of a specific gene/pathway in the complex gut-microbiota – host interactions by using knock-out models.
- Mouse models are inbred, providing a homogenous genetic background, a cleaner system to dissect signals from gut-bacteria–host interactions and improve reproducibility of experiments.
- Sources of variations such as diets and housing conditions are generally controlled for in experiments, limiting unwanted influences of noises from the surrounding environment to gut microbiota.

**Limitations**

- Despite important similarities, mice are different from humans in anatomy, genetics and physiology, and thus mouse models *cannot* fully recapitulate human systems.
- Different mouse models can give rise to diverged shifts in gut microbiota composition.
- Cross-talk between gut microbiota and the host is host-specific so observations in mouse models might not be applicable in humans.
- Genetic homogeneity implies that the inbred mouse strains cannot capture the inherent genetic variations in the human population.
- Multiple factors, such as genetic background, birth mode of delivery (caesarean or vaginal), mode of feeding (breast or bottle), diet, medical history and social activities all contribute to shaping a ‘real-life’ gut microbiota in humans. Absence of these factors in mice implies that gut microbiota in mouse models cannot reflect a ‘real-life’ gut microbiota.

### Genetic background and founder effects

A wide range of experiments in which gene-knockout mouse models have been created, yielding different genetic backgrounds, have highlighted that genetics is an important determinant of gut microbiota composition (Benson et al., 2010; Hildebrand et al., 2013; Kovacs et al., 2011). In experiments using wild-type inbred mouse strains, genetic background effects have been suggested to be stronger than gender (Hildebrand et al., 2013; Kovacs et al., 2011) but to contribute less than environmental (cage) factors and stochastic effects to variation in gut microbiota composition (Hildebrand et al., 2013). One explanation for the influence of genetic background is the variation in the efficacy of colonization by bacteria on hosts with different genetic backgrounds. For example, Wos-Oxley et al. found that Clostridiales bacteria colonize the gut of humanized rats more efficiently than that of humanized mice (Wos-Oxley et al., 2012).

In humans, the role of genetics in gut microbiota composition has been scrutinized in twin studies. A study by Turnbaugh and colleagues shows that monozygotic twins are less dissimilar in terms of gut microbiota composition within twin pairs than pairs of dizygotic twins, and that the gut microbiota of twins are more similar to each other than to their mothers’ (Turnbaugh et al., 2009a). Given the similar environment that the twins share with each other and with their mother, it is obvious that the more closely related they are in genetics, the more similar the gut microbiota composition becomes.

Murine models are usually inbred in order to reduce the phenotypic variation that arises from genetic heterogeneity. Controlling genetic background offers opportunities to disentangle complex relationships between host genetics and gut microbiota, and potentially to elucidate the mechanisms of host-microbiota interaction. Knock-out mouse models, for example, are crucial tools for functional experiments on gut pathologies. For instance, the study of Muc2 knock-out mice, which lack Muc2, the most abundant secreted gastrointestinal mucin, led to the discovery of the link between this protein and suppression of colorectal cancer (Velcich et al., 2002). Nevertheless, model knock-out work is not without pitfalls either. Genetic homogeneity, which fails to portray inter-individual variations in humans, can pose problems when translating the results of studies using inbred mouse strains to humans in gut microbiota research. For instance, results from the IL-10 knock-out mouse model implied that this anti-inflammatory cytokine plays an important role in controlling IBD. However, clinical trials of IL-10 treatment on IBD in humans yielded modest results (Fedorak et al., 2000; Schreiber et al., 2000). One of the explanations for the minimal results was the unexpected heterogeneity in IL-10 receptor pool in humans as compared with mice (Barnett and Fraser, 2011). This heterogeneity might influence the efficiency of IL-10 in dampening inflammation via interactions with target cells that express IL-10 receptors. Similarly, variations in drug efficacy due to genetic polymorphism in human population are another important issue that was discussed (Ma and Lu, 2011). However, the lack of genetic background diversity in inbred mice could be solved in experimental setups by using outbred strains or wild mice, or genetically modified models from multiple mouse strains, in order to partially mimic the genetic diversity of the human population. Another advantage of this approach is that the maternal effect is minimized when mice are all from different mothers.

Given the complex interactions of gut microbiota with the host and the surrounding environment, and stochastic effects, which still largely contribute to gut microbiota variations (Hildebrand et al., 2013), it is crucial to control for all contributing factors to increase statistical power. These include genetic background, cage effects, maternal effects, diet, treatments and the number of biological replicates. In addition, for the reproducibility of gut microbiota studies, standardization of protocols for animal handling as well as sample storage and processing and data analysis are of utmost importance.

## The limitations of certain mouse models

Given its involvement in a wide range of physiological processes, the gut microbiota inevitably plays an important role in maintaining host health and wellbeing. The number of associations between gut microbiota composition and function and host diseases is increasing, with such diseases including obesity, IBD, diabetes and allergic diseases, such as asthma (Russell et al., 2012) and atopy (Lee et al., 2014). These chronic diseases place a substantial financial burden on the healthcare systems of developed societies (Burisch et al., 2013; Meng et al., 2014; Yang and Zhang, 2014). These pathologies were already known to be triggered by multiple genetic and environmental factors. In addition, the discovery of a contribution of the gut microbiota to their development has added another layer of complexity to understanding their pathophysiological mechanisms. Although the association of gut microbiota with a disease does not imply causality, understanding its role does open up potentially new opportunities to treat or mitigate the disease. Employing mouse models to study host-microbe interactions allows functional experimentation to dissect underlying mechanisms that are not always possible in humans. On the other hand, mouse models also have their limitiations (Box 2). Here, we illustrate the limitations of mouse disease models from a microbiota perspective, focusing on models of colitis as well as on models in which germ-free mice are inoculated with human gut microbiota samples (humanized gnotobiotic mice) (Table 2).

**Table 2.**
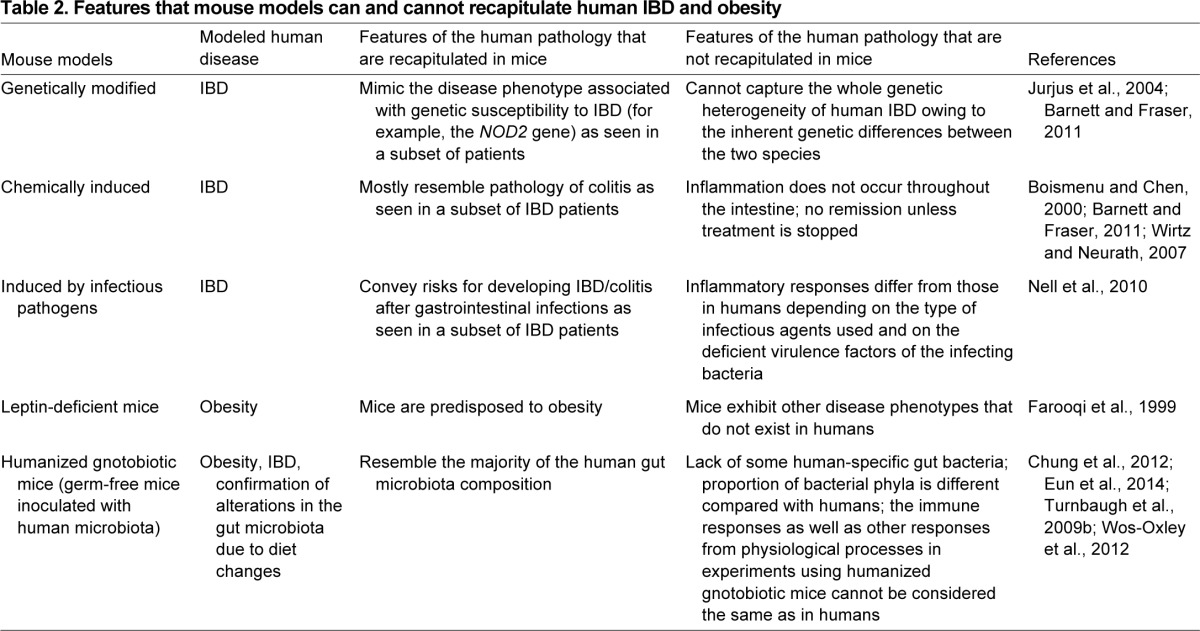
Features that mouse models can and cannot recapitulate human IBD and obesity

### Genetically modified and/or chemically induced models

Genetically modified murine models are powerful tools for studying the pathophysiological mechanisms of human diseases, and are now increasingly used to study the complex interactions between the gut microbiota and host in normal homeostasis and disease. However, targeted genes are often involved in multiple pathways, confounding the inferences that can be made about the association between the gene expression and gut microbiota composition and function. Moreover, many mouse models do not recapitulate exactly the modeled human disease (Table 2), with each model having different limitations that need to be taken into account to translate the results to humans. A typical example is colitis, for which there are currently about 60 different mouse models (Peloquin and Nguyen, 2013; Wirtz and Neurath, 2007).

IBD is a complex disease, proposed to be an autoimmune disorder with the involvement of multiple genetic loci as well as a contribution of the gut microbiota in the development of the disease. IBD research has yielded several mouse models (usually referred to as colitis models because the pathology mostly resembles ulcerative colitis symptoms, as mentioned above), including genetically modified models, where a candidate gene involved in IBD development is altered or knocked out, and models in which chemicals or infectious pathogens are used to induce gut inflammation and changes in gut microbiota composition, leading to colitis. Despite the numerous mouse models developed for IBD and their contributions to our knowledge of the underlying mechanisms of the disease, none of them fully mimic the pathophysiology of human IBD (Peloquin and Nguyen, 2013; Wirtz and Neurath, 2007) (Table 2). However, these genetically modified models can resemble pretty well the phenotype of a subset of individuals with IBD who carry genetic defects and thus are predisposed for developing IBD (Table 2). On the other hand, most of the models show inflammation/lesions in the large, instead of the small, intestine, which more closely resembles ulcerative colitis symptoms (Peloquin and Nguyen, 2013). Moreover, the development of colitis in mouse models has been shown to be affected by the genetic background of the used strain [i.e. some strains are intrinsically more susceptible to developing colitis (Büchler et al., 2012)] and the interactions with the gut microbiota [e.g. some models do not develop colitis in germ-free conditions (Hudcovic et al., 2001)]. The concern is not only that symptoms are not concordant with human IBD, but that the underlying mechanism, such as the cross-talk between the immune system and the gut microbiota, might not be the same in the mouse model being used.

Another common way to induce colitis in mice is by using chemical agents, such as DSS, to damage gut epithelial cells, or molecules to stimulate an immune response in the colonic mucosa (e.g. haptens, such as TNBS and oxazolone). These agents induce either acute or chronic intestinal inflammation in mice, leading to the development of colitis. Although widely used, variations exist in experimental protocols and in the mice housing conditions, sometimes giving rise to differing results in experiments conducted on the same mouse model. For instance, Kitajima et al. observed that germ-free laboratory mice are very susceptible to DSS-induced colitis, whereas conventionally housed wild-type laboratory mice are more tolerant (Kitajima et al., 2002). This result contradicts the observation that germ-free mice, either immune-deficient or immune-competent, are resistant to DSS-induced colitis as compared with their respective conventionally housed mice, as reported in the study by Hudcovic et al. (Hudcovic et al., 2001). Another example of inconsistencies from chemically induced models of colitis is from the diverging results of the studies by Fayad et al. (Fayad et al., 2007) and Nishihara et al. (Nishihara et al., 2006). In the former, the adiponectin knock-out mice did not develop colitis when administered with either DSS or TNBS, whereas, in the latter, the DSS-treated adiponectin knock-out mice developed more severe colitis compared with wild-type mice. The discrepancies in outcomes of these studies raise concerns for the use of these models in IBD and colitis research. Furthermore, the chemical itself might affect the composition or function of the resident gut microbiota before the onset of colitis (Nagalingam et al., 2011). In support of this proposition, DSS has recently been shown to alter the protein expression profiles of gut-resident *E. coli* in DSS-treated mice (Schumann et al., 2012). These cases highlight how the use of colitis mouse models in gut microbiota research will require controlling for additional variables beyond the common confounders (such as host genetic background and environmental factors). Therefore, the interpretation of results from colitis mouse models, at least from the microbiota perspective, should be done with caution. The intrinsic complexity of gut microbiota research, together with the inconsistency of colitis mouse models (chemically induced or genetically engineered), makes interpreting the results of gut microbiota research from these models in terms of human disease far from easy.

### Humanized gnotobiotic mice

Humanized gnotobiotic mice, which result from the inoculation of a human gut microbiota sample in germ-free mice, provide a powerful tool for gut microbiota studies because these models can recapitulate a large part of the human gut microbiota phylogenetic composition (100% of phyla, 11/12 classes and ~ 88% of genus-level taxa). This approach has been widely employed in many studies because it allows perturbations in a ‘human-like system’ and is considered to be the gold standard for confirming associations and trying to prove causality in gut microbiota research (Faith et al., 2011; Goodman et al., 2011; McNulty et al., 2011; Smith et al., 2013a; Turnbaugh et al., 2009b). However, it should be highlighted that the host-microbe relationships in these humanized models do not necessarily reflect the real relationships seen in humans, because the gut microbiota is transplanted into a host with which it has not co-evolved. Turnbaugh et al. reported that some resident bacterial taxa in the human gut microbiota are absent in the humanized mouse gut microbiota (Turnbaugh et al., 2009b). Given the low abundance of these bacteria, their absence is generally considered to be less important for the balance and functions of the gut microbiota. However, the complex interactions between the bacteria composing the gut community (Faust and Raes, 2012) suggest that this might not be the case. Furthermore, low-abundance microbial species can be of essential importance to the ecosystem, as shown by a recent study in which the immune system of humanized mice did not mature normally (Chung et al., 2012). This study shows that the gut microbiota composition of humanized mice in general does not differ significantly from that of the initial human donors, except for the absence of many low-abundance Firmicutes, but these mice fail to recapitulate a comprehensive response to infections. Moreover, as discussed above, gnotobiotic mouse phenotypic responses can vary in different recipient germ-free mouse genetic backgrounds. Overall, it has been found that even though gnotobiotic mice are being increasingly used as models for studying the human gut microbiota, they might not fully recapitulate the mechanisms of the human-host–gut-microbiota interaction. This said, they are one of very few methods to assess causality in microbiota research, and thus further development and improvement of this approach is essential.

In summary, mouse models are a powerful tool in gut microbiota research, and offer the possibility to perform experiments that would be too invasive for human subjects and with better control over confounding factors. Despite the various drawbacks of mouse models for colitis, these models have provided valuable insights into colitis and the physiopathology of IBD, identifying factors that trigger the progression of this complex disease (Barnett and Fraser, 2011; Nell et al., 2010; Peloquin and Nguyen, 2013; Uhlig and Powrie, 2009). Similarly, although the gap between humanized mouse models and the human gut microbiota within its original host needs to be acknowledged, these models provide unique possibilities to manipulate the human microbiota and potentially assess causality in the role of the gut microbiota in health and disease. Recently, a study by Ridaura et al. showed that obesity phenotypes can be transferred from human to humanized mice and between co-housed mice (Ridaura et al., 2013). Such studies are extremely valuable for understanding the underlying disease mechanisms and causative agents, and thus for getting closer to developing preventive or therapeutic treatments.

## Outlook: alternatives to mice

The strengths and limitations of mice as models for gut microbiota research are clear, but are there better alternatives for the research community to develop or consider? Some studies have been exploring the gut microbiota of other animal models, such as rats (Alpert et al., 2008; Liou et al., 2013; Wos-Oxley et al., 2012), dogs (Middelbos et al., 2010), pigs (Lamendella et al., 2011; Riboulet-Bisson et al., 2012) and primates, such as chimpanzees (Moeller et al., 2012) and macaques (McKenna et al., 2008). Rats are proposed to be more representative of the human gut microbiota than mice because the gut bacterial communities of humanized rats more closely reflect the gut microbiota of human donors (Wos-Oxley et al., 2012). Specifically, humanized rat models have a more similar Firmicutes:Bacteroidetes ratio to human donors and could represent the human donor better than could germ-free mouse models. This difference is thought to be due to the fact that the rat has a microbiota that is more similar to humans than mice do, thus facilitating the establishment of the inoculated microbiota (Wos-Oxley et al., 2012). Furthermore, it was hypothesized that the differences between mice and rats in recapitulating the human donors’ microbiota is due to the intrinsic differences in genetic backgrounds of the recipient models, which might control for the establishment of the transferred microbiota (Wos-Oxley et al., 2012).

Another non-murine rodent model that has been employed in gut microbiota research is the guinea pig. Given the homology that exists between human and guinea pig E-cadherin, a transmembrane adhesion protein that locates to the intestinal surface, this animal has become an important model for studying infection by the human pathogen *Listeria monocytogenes*. Because a healthy commensal intestinal microbiota affects the potential for infection by a pathogen (pathogen exclusion), the gut microbiota of guinea pigs has been investigated in several studies (Hildebrand et al., 2012; Yanabe et al., 2001). For example, in the study by Yanabe et al., germ-free guinea pigs were inoculated with combinations of cecal content from conventional guinea pigs to determine efficient cocktails to establish a healthy SPF colony (Yanabe et al., 2001). The authors found that the transferred microbiota became similar to the gut microbiota of conventional guinea pigs and stayed stable over a long period. More recently, another study employed metagenomic approaches to compare the gut microbiota composition of humans and guinea pigs (Hildebrand et al., 2012). The study revealed that humans and guinea pigs share dominant bacterial phyla, but the abundance of these common phyla (22 out of 26 phyla) is significantly higher in guinea pigs as compared to humans. In addition, significant differences in abundance were found across 320 out of 376 genera. Functional categories, such as metabolism or cell membrane biogenesis, also differ significantly in abundance between the human and guinea pig gut microbiome (Hildebrand et al., 2012).

The canine gut microbiota has also been investigated in several studies, given the similarity in gut morphology and functioning to the digestive system of humans (Middelbos et al., 2010; Suchodolski et al., 2008; Suchodolski et al., 2009). Recently, a metagenomic study described the composition (bacteria, archaea and fungi) and function of the dog microbiome (Swanson et al., 2011). The canine microbiota has similar dominant phyla (Bacteroidetes and Firmicutes) to humans. However, at the genus level, the canine gut microbiota is distinct by a greater prevalence of the genus *Fusobacteria* than in humans. The dog gut microbiota also has a higher Fibrobacteres and Acidobacteria abundance and lower abundance of Actinobacteria than humans. Clustering of fecal microbiomes on the basis of metabolic capacity of gut microbial communities of canines, humans and mice showed that the canine microbiome clustered more closely to the human than did the mouse microbiome (Swanson et al., 2011).

One potential competitor for mouse as a prime microbiome research model is pig. The pig gut microbiota is becoming an increasingly intensive area of study owing to the scale of the pig husbandry industry, as well as to the similarities in anatomy, physiology and immunology to the human gastrointestinal tract (Litten-Brown et al., 2010). Swine gut microbiota has been investigated in several studies, especially involving antibiotic interventions (Kim et al., 2012), which are of industrial relevance. An in-depth study of swine gut microbiota composition in the Yorkshire pig breed showed that human and pig microbiota shared similar diversity patterns, with the two dominant phyla in pigs also being Bacteroidetes and Firmicutes (Lamendella et al., 2011). At the genus level, the swine gut microbiota harbors more Spirochaetes and *Prevotella* than the human gut microbiota. Interestingly, *Anaerovibrio* and *Treptonema* are genera exclusive to pig fecal metagenomes (Lamendella et al., 2011). Miniature pigs are becoming promising models for biomedical research owing to their small size. In general, miniature pigs become obese when fed *ad libitum* and are therefore employed as models for obesity and metabolic syndrome. In a study, in which Ossabaw and Yucatan miniature pig breeds were assessed for their suitability as models of metabolic syndrome and coronary artery disease, it was indicated that Ossabaw miniature pig is a superior model (Neeb et al., 2010). Likewise, the gut microbiota composition of two miniature-pig breeds, Gottingen and Ossabaw, was investigated for their responses to obesity induction (Pedersen et al., 2013). The study showed that the major phyla of miniature pig gut microbiota are Firmicutes and Bacteroidetes. Interestingly, the two miniature pig breeds responded differently to an obesity-inducing diet: Ossabaw gut microbiota displayed more of the characteristics of a ‘healthy’ obese microbiota, whereas Gottigen gut microbiota had changes similar to metabolic syndrome, such as those found in the gut microbiota profiles of type 2 diabetic mice, again emphasizing the importance of genetic background in gut microbiota response to perturbation.

Non-human primates are also suitable models for human gut microbiota research, with the obvious advantage of close evolutionary relatedness to humans. Interestingly, chimpanzees were found to harbor three enterotypes and these had a similar composition to those found in humans (Arumugam et al., 2011; Moeller et al., 2012), suggesting a conservation of the stratification of microbial communities in chimpanzees and humans (Moeller and Ochman, 2014). By contrast, studies investigating the gut microbiota composition of macaques showed that the community of gut bacteria in these primates is quite different to that of humans or mice (McKenna et al., 2008). Specifically, the macaque gut microbiota clearly separated from that of human and mice in a principal coordinate analysis on microbiota phylogenetic composition. One of the distinct features of the macaque gut microbiota is the presence and abundance of *Treponema*, a genus from the phylum Spirochaetes. By contrast, *Bacteroides*, one of the most abundant genera in human gut microbiota, is found to be rare in this species (McKenna et al., 2008). Despite the advantage of close evolutionary relatedness and physiology to humans, primate models have more stringent ethical restrictions for experimentation and breeding/care difficulties compared with mice.

## Conclusions

The pioneering studies cited above on new animal models for gut microbiota research have greatly demonstrated their potential. However, despite the limitations of mouse models outlined in this review, their advantages are numerous and, furthermore, the amount of research and knowledge on mouse gastroenterology, genetics and immunology far surpasses any other model. Murine mouse models provide a range of customizable genotypes and phenotypes far superior to any other model organism. They have thus played a very important role in the emerging gut microbiota research field. Owing to their widespread use in biomedical research, these models are complemented with extensive knowledge on genetic background and deep phenotypic and functional characterization. Moreover, with well-set-up standardized mouse house facilities throughout labs in the world, conducting experiments on mouse models, even germ-free ones, can be more easily achieved than with other models.

Each one of the animal models referred to here shows some similarity to the physiology of the human digestive system, thus providing useful knowledge from different angles about the gut microbiota in health and disease. It is clear that information obtained from studies using alternative models has diversified our understanding of the mammalian gut microbiota in general and has deepened our knowledge of each model separately. It is, however, important to keep in mind that models always have some degree of dissimilarity with the system modeled. Therefore, results from animal models, including the popular murine ones, are not always translatable to humans and conclusions should be made with caution. In addition, even well-controlled gut microbiota experiments using mouse models show important inter-study variations due to confounding factors in the experimental setup, such as mouse house origin, maternal effects, environmental conditions (food composition, light, stress factors, pathogen infection), genetic backgrounds and in the downstream analysis methods applied. There have been recent efforts to standardize gut microbiota experiments, for example by establishing standardized microbiota in isobiotic mice that would be shared by institutions involved in gut microbiota research (Hooper et al., 2012). Although these efforts are still in their infancy (Würbel, 2000), they will increase result reproducibility and inter-study comparability, and allow for the healthy growth of the gut microbiota research field.

## Supplementary Material

Supplementary Material

## References

[b1-0080001] Akaza H. (2012). Prostate cancer chemoprevention by soy isoflavones: role of intestinal bacteria as the “second human genome”. Cancer Sci. 103, 969–975. 22372745 10.1111/j.1349-7006.2012.02257.x PMC7685082

[b2-0080001] Alpert C. Sczesny S. Gruhl B. Blaut M. (2008). Long-term stability of the human gut microbiota in two different rat strains. Curr. Issues Mol. Biol. 10, 17–24. 18525103

[b3-0080001] Andoh A. Kuzuoka H. Tsujikawa T. Nakamura S. Hirai F. Suzuki Y. Matsui T. Fujiyama Y. Matsumoto T. (2012). Multicenter analysis of fecal microbiota profiles in Japanese patients with Crohn’s disease. J. Gastroenterol. 47, 1298–1307. 22576027 10.1007/s00535-012-0605-0

[b4-0080001] Arumugam M. Raes J. Pelletier E. Le Paslier D. Yamada T. Mende D. R. Fernandes G. R. Tap J. Bruls T. Batto J.-M. MetaHIT Consortium (2011). Enterotypes of the human gut microbiome. Nature 473, 174–180. 21508958 10.1038/nature09944 PMC3728647

[b5-0080001] Bäckhed F. Manchester J. K. Semenkovich C. F. Gordon J. I. (2007). Mechanisms underlying the resistance to diet-induced obesity in germ-free mice. Proc. Natl. Acad. Sci. USA 104, 979–984. 17210919 10.1073/pnas.0605374104 PMC1764762

[b6-0080001] Barnett M. Fraser A. (2011). Animal models of colitis: lessons learned, and their relevance to the clinic. In Ulcerative Colitis – Treatments, Special Populations and the Future (ed. O’Connor M. Dr). Rijeka, Croatia: InTech.

[b7-0080001] Benson A. K. Kelly S. A. Legge R. Ma F. Low S. J. Kim J. Zhang M. Oh P. L. Nehrenberg D. Hua K. (2010). Individuality in gut microbiota composition is a complex polygenic trait shaped by multiple environmental and host genetic factors. Proc. Natl. Acad. Sci. USA 107, 18933–18938. 20937875 10.1073/pnas.1007028107 PMC2973891

[b8-0080001] Bercik P. Collins S. M. Verdu E. F. (2012). Microbes and the gut-brain axis. Neurogastroenterol. Motil. 24, 405–413. 22404222 10.1111/j.1365-2982.2012.01906.x

[b9-0080001] Berry D. Schwab C. Milinovich G. Reichert J. Ben Mahfoudh K. Decker T. Engel M. Hai B. Hainzl E. Heider S. (2012). Phylotype-level 16S rRNA analysis reveals new bacterial indicators of health state in acute murine colitis. ISME J. 6, 2091–2106. 22572638 10.1038/ismej.2012.39 PMC3475367

[b10-0080001] Blanchard R. J. Blanchard D. C. Agullana R. Weiss S. M. (1991). Twenty-two kHz alarm cries to presentation of a predator, by laboratory rats living in visible burrow systems. Physiol. Behav. 50, 967–972. 1805287 10.1016/0031-9384(91)90423-l

[b11-0080001] Boismenu R. Chen Y. (2000). Insights from mouse models of colitis. J. Leukoc. Biol. 67, 267–278. 10733087 10.1002/jlb.67.3.267

[b12-0080001] Brinkman B. M. Hildebrand F. Kubica M. Goosens D. Del Favero J. Declercq W. Raes J. Vandenabeele P. (2011). Caspase deficiency alters the murine gut microbiome. Cell Death Dis. 2, e220. 22012254 10.1038/cddis.2011.101 PMC3219086

[b13-0080001] Brinkman B. M. Becker A. Ayiseh R. B. Hildebrand F. Raes J. Huys G. Vandenabeele P. (2013). Gut microbiota affects sensitivity to acute DSS-induced colitis independently of host genotype. Inflamm. Bowel Dis. 19, 2560–2567. 24105395 10.1097/MIB.0b013e3182a8759a

[b14-0080001] Büchler G. Wos-Oxley M. L. Smoczek A. Zschemisch N.-H. Neumann D. Pieper D. H. Hedrich H. J. Bleich A. (2012). Strain-specific colitis susceptibility in IL10-deficient mice depends on complex gut microbiota-host interactions. Inflamm. Bowel Dis. 18, 943–954. 22238116 10.1002/ibd.21895

[b15-0080001] Burisch J. Jess T. Martinato M. Lakatos P. L. ECCO -EpiCom (2013). The burden of inflammatory bowel disease in Europe. J. Crohn’s Colitis 7, 322–337. 23395397 10.1016/j.crohns.2013.01.010

[b16-0080001] Casteleyn C. Rekecki A. Van der Aa A. Simoens P. Van den Broeck W. (2010). Surface area assessment of the murine intestinal tract as a prerequisite for oral dose translation from mouse to man. Lab. Anim. 44, 176–183. 20007641 10.1258/la.2009.009112

[b17-0080001] Catrysse L. Vereecke L. Beyaert R. van Loo G. (2014). A20 in inflammation and autoimmunity. Trends Immunol. 35, 22–31. 24246475 10.1016/j.it.2013.10.005

[b18-0080001] Cho I. Yamanishi S. Cox L. Methé B. A. Zavadil J. Li K. Gao Z. Mahana D. Raju K. Teitler I. (2012). Antibiotics in early life alter the murine colonic microbiome and adiposity. Nature 488, 621–626. 22914093 10.1038/nature11400 PMC3553221

[b19-0080001] Chung H. Pamp S. J. Hill J. A. Surana N. K. Edelman S. M. Troy E. B. Reading N. C. Villablanca E. J. Wang S. Mora J. R. (2012). Gut immune maturation depends on colonization with a host-specific microbiota. Cell 149, 1578–1593. 22726443 10.1016/j.cell.2012.04.037 PMC3442780

[b20-0080001] Cotillard A. Kennedy S. P. Kong L. C. Prifti E. Pons N. Le Chatelier E. Almeida M. Quinquis B. Levenez F. Galleron N. ANR MicroObes consortium (2013). Dietary intervention impact on gut microbial gene richness. Nature 500, 585–588. 23985875 10.1038/nature12480

[b21-0080001] Cryan J. F. O’Mahony S. M. (2011). The microbiome-gut-brain axis: from bowel to behavior. Neurogastroenterol. Motil. 23, 187–192. 21303428 10.1111/j.1365-2982.2010.01664.x

[b22-0080001] Cunliffe R. N. Rose F. R. Keyte J. Abberley L. Chan W. C. Mahida Y. R. (2001). Human defensin 5 is stored in precursor form in normal Paneth cells and is expressed by some villous epithelial cells and by metaplastic Paneth cells in the colon in inflammatory bowel disease. Gut 48, 176–185. 11156637 10.1136/gut.48.2.176 PMC1728187

[b23-0080001] De Angelis M. Piccolo M. Vannini L. Siragusa S. De Giacomo A. Serrazzanetti D. I. Cristofori F. Guerzoni M. E. Gobbetti M. Francavilla R. (2013). Fecal microbiota and metabolome of children with autism and pervasive developmental disorder not otherwise specified. PLoS ONE 8, e76993. 24130822 10.1371/journal.pone.0076993 PMC3793965

[b24-0080001] De Filippo C. Cavalieri D. Di Paola M. Ramazzotti M. Poullet J. B. Massart S. Collini S. Pieraccini G. Lionetti P. (2010). Impact of diet in shaping gut microbiota revealed by a comparative study in children from Europe and rural Africa. Proc. Natl. Acad. Sci. USA 107, 14691–14696. 20679230 10.1073/pnas.1005963107 PMC2930426

[b25-0080001] Degen G. H. Janning P. Diel P. Bolt H. M. (2002). Estrogenic isoflavones in rodent diets. Toxicol. Lett. 128, 145–157. 11869825 10.1016/s0378-4274(02)00009-7

[b26-0080001] DeVries A. C. Craft T. K. S. Glasper E. R. Neigh G. N. Alexander J. K. (2007). 2006 Curt P. Richter award winner: Social influences on stress responses and health. Psychoneuroendocrinology 32, 587–603. 17590276 10.1016/j.psyneuen.2007.04.007

[b27-0080001] Ding T. Schloss P. D. (2014). Dynamics and associations of microbial community types across the human body. Nature 509, 357–360. 24739969 10.1038/nature13178 PMC4139711

[b28-0080001] Duboc H. Rajca S. Rainteau D. Benarous D. Maubert M.-A. Quervain E. Thomas G. Barbu V. Humbert L. Despras G. (2013). Connecting dysbiosis, bile-acid dysmetabolism and gut inflammation in inflammatory bowel diseases. Gut 62, 531–539. 22993202 10.1136/gutjnl-2012-302578

[b29-0080001] Duncan S. H. Lobley G. E. Holtrop G. Ince J. Johnstone A. M. Louis P. Flint H. J. (2008). Human colonic microbiota associated with diet, obesity and weight loss. Int. J. Obes. 32, 1720–1724. 10.1038/ijo.2008.155 18779823

[b30-0080001] Eckburg P. B. Bik E. M. Bernstein C. N. Purdom E. Dethlefsen L. Sargent M. Gill S. R. Nelson K. E. Relman D. A. (2005). Diversity of the human intestinal microbial flora. Science 308, 1635–1638. 15831718 10.1126/science.1110591 PMC1395357

[b31-0080001] Elinav E. Strowig T. Kau A. L. Henao-Mejia J. Thaiss C. A. Booth C. J. Peaper D. R. Bertin J. Eisenbarth S. C. Gordon J. I. (2011). NLRP6 inflammasome regulates colonic microbial ecology and risk for colitis. Cell 145, 745–757. 21565393 10.1016/j.cell.2011.04.022 PMC3140910

[b32-0080001] Eun C. S. Mishima Y. Wohlgemuth S. Liu B. Bower M. Carroll I. M. Sartor R. B. (2014). Induction of bacterial antigen-specific colitis by a simplified human microbiota consortium in gnotobiotic interleukin-10−/− mice. Infect. Immun. 82, 2239–2246. 24643531 10.1128/IAI.01513-13 PMC4019192

[b33-0080001] Faith J. J. McNulty N. P. Rey F. E. Gordon J. I. (2011). Predicting a human gut microbiota’s response to diet in gnotobiotic mice. Science 333, 101–104. 21596954 10.1126/science.1206025 PMC3303606

[b34-0080001] Farooqi I. S. Jebb S. A. Langmack G. Lawrence E. Cheetham C. H. Prentice A. M. Hughes I. A. McCamish M. A. O’Rahilly S. (1999). Effects of Recombinant Leptin Therapy in a Child with Congenital Leptin Deficiency. N. Engl. J. Med. 341, 879–884. 10486419 10.1056/NEJM199909163411204

[b35-0080001] Faust K. Raes J. (2012). Microbial interactions: from networks to models. Nat. Rev. Microbiol. 10, 538–550. 22796884 10.1038/nrmicro2832

[b36-0080001] Fayad R. Pini M. Sennello J. A. Cabay R. J. Chan L. Xu A. Fantuzzi G. (2007). Adiponectin deficiency protects mice from chemically induced colonic inflammation. Gastroenterology 132, 601–614. 17258715 10.1053/j.gastro.2006.11.026

[b37-0080001] Fedorak R. N. Gangl A. Elson C. O. Rutgeerts P. Schreiber S. Wild G. Hanauer S. B. Kilian A. Cohard M. LeBeaut A. (2000). Recombinant human interleukin 10 in the treatment of patients with mild to moderately active Crohn’s disease. The Interleukin 10 Inflammatory Bowel Disease Cooperative Study Group. Gastroenterology 119, 1473–1482. 11113068 10.1053/gast.2000.20229

[b38-0080001] Fernandez-Raudales D. Hoeflinger J. L. Bringe N. A. Cox S. B. Dowd S. E. Miller M. J. Gonzalez de Mejia E. (2012). Consumption of different soymilk formulations differentially affects the gut microbiomes of overweight and obese men. Gut Microbes 3, 490–500. 22895080 10.4161/gmic.21578 PMC3495786

[b39-0080001] Friswell M. K. Gika H. Stratford I. J. Theodoridis G. Telfer B. Wilson I. D. McBain A. J. (2010). Site and strain-specific variation in gut microbiota profiles and metabolism in experimental mice. PLoS ONE 5, e8584. 20052418 10.1371/journal.pone.0008584 PMC2798964

[b40-0080001] Galley J. D. Nelson M. C. Yu Z. Dowd S. E. Walter J. Kumar P. S. Lyte M. Bailey M. T. (2014). Exposure to a social stressor disrupts the community structure of the colonic mucosa-associated microbiota. BMC Microbiol. 14, 189. 25028050 10.1186/1471-2180-14-189 PMC4105248

[b41-0080001] Ghosh D. Porter E. Shen B. Lee S. K. Wilk D. Drazba J. Yadav S. P. Crabb J. W. Ganz T. Bevins C. L. (2002). Paneth cell trypsin is the processing enzyme for human defensin-5. Nat. Immunol. 3, 583–590. 12021776 10.1038/ni797

[b42-0080001] Goodman A. L. Kallstrom G. Faith J. J. Reyes A. Moore A. Dantas G. Gordon J. I. (2011). Extensive personal human gut microbiota culture collections characterized and manipulated in gnotobiotic mice. Proc. Natl. Acad. Sci. USA 108, 6252–6257. 21436049 10.1073/pnas.1102938108 PMC3076821

[b43-0080001] Hildebrand F. Ebersbach T. Nielsen H. B. Li X. Sonne S. B. Bertalan M. Dimitrov P. Madsen L. Qin J. Wang J. (2012). A comparative analysis of the intestinal metagenomes present in guinea pigs (Cavia porcellus) and humans (Homo sapiens). BMC Genomics 13, 514. 23020652 10.1186/1471-2164-13-514 PMC3472315

[b44-0080001] Hildebrand F. Nguyen T. L. Brinkman B. Yunta R. G. Cauwe B. Vandenabeele P. Liston A. Raes J. (2013). Inflammation-associated enterotypes, host genotype, cage and inter-individual effects drive gut microbiota variation in common laboratory mice. Genome Biol. 14, R4. 23347395 10.1186/gb-2013-14-1-r4 PMC4053703

[b45-0080001] Hooper L. V. Littman D. R. Macpherson A. J. (2012). Interactions between the microbiota and the immune system. Science 336, 1268–1273. 22674334 10.1126/science.1223490 PMC4420145

[b46-0080001] Hudcovic T. Stĕpánková R. Cebra J. Tlaskalová-Hogenová H. (2001). The role of microflora in the development of intestinal inflammation: acute and chronic colitis induced by dextran sulfate in germ-free and conventionally reared immunocompetent and immunodeficient mice. Folia Microbiol. (Praha) 46, 565–572. 11898350 10.1007/BF02818004

[b47-0080001] Human Microbiome Project Consortium (2012). Structure, function and diversity of the healthy human microbiome. Nature 486, 207–214. 22699609 10.1038/nature11234 PMC3564958

[b48-0080001] Ivanov I. I. Atarashi K. Manel N. Brodie E. L. Shima T. Karaoz U. Wei D. Goldfarb K. C. Santee C. A. Lynch S. V. (2009). Induction of intestinal Th17 cells by segmented filamentous bacteria. Cell 139, 485–498. 19836068 10.1016/j.cell.2009.09.033 PMC2796826

[b49-0080001] Jacoby R. O. Lindsey J. R. (1998). Risks of infection among laboratory rats and mice at major biomedical research institutions. ILAR J. 39, 266–271. 11528087 10.1093/ilar.39.4.266 PMC7537657

[b50-0080001] Joossens M. Huys G. Cnockaert M. De Preter V. Verbeke K. Rutgeerts P. Vandamme P. Vermeire S. (2011). Dysbiosis of the faecal microbiota in patients with Crohn’s disease and their unaffected relatives. Gut 60, 631–637. 21209126 10.1136/gut.2010.223263

[b51-0080001] Jostins L. Ripke S. Weersma R. K. Duerr R. H. McGovern D. P. Hui K. Y. Lee J. C. Schumm L. P. Sharma Y. Anderson C. A. International IBD Genetics Consortium (IIBDGC) (2012). Host-microbe interactions have shaped the genetic architecture of inflammatory bowel disease. Nature 491, 119–124. 23128233 10.1038/nature11582 PMC3491803

[b52-0080001] Jurjus A. R. Khoury N. N. Reimund J.-M. (2004). Animal models of inflammatory bowel disease. J. Pharmacol. Toxicol. Methods 50, 81–92. 15385082 10.1016/j.vascn.2003.12.002

[b53-0080001] Kang D.-W. Park J. G. Ilhan Z. E. Wallstrom G. Labaer J. Adams J. B. Krajmalnik-Brown R. (2013). Reduced incidence of Prevotella and other fermenters in intestinal microflora of autistic children. PLoS ONE 8, e68322. 23844187 10.1371/journal.pone.0068322 PMC3700858

[b54-0080001] Kellermayer R. Dowd S. E. Harris R. A. Balasa A. Schaible T. D. Wolcott R. D. Tatevian N. Szigeti R. Li Z. Versalovic J. (2011). Colonic mucosal DNA methylation, immune response, and microbiome patterns in Toll-like receptor 2-knockout mice. FASEB J. 25, 1449–1460. 21228220 10.1096/fj.10-172205 PMC3079304

[b55-0080001] Khor B. Gardet A. Xavier R. J. (2011). Genetics and pathogenesis of inflammatory bowel disease. Nature 474, 307–317. 21677747 10.1038/nature10209 PMC3204665

[b56-0080001] Kim H. B. Borewicz K. White B. A. Singer R. S. Sreevatsan S. Tu Z. J. Isaacson R. E. (2012). Microbial shifts in the swine distal gut in response to the treatment with antimicrobial growth promoter, tylosin. Proc. Natl. Acad. Sci. USA 109, 15485–15490. 22955886 10.1073/pnas.1205147109 PMC3458334

[b57-0080001] Kitajima S. Morimoto M. Sagara E. (2002). A model for dextran sodium sulfate (DSS)-induced mouse colitis: bacterial degradation of DSS does not occur after incubation with mouse cecal contents. Exp. Anim. 51, 203–206. 12012734 10.1538/expanim.51.203

[b58-0080001] Koren O. Knights D. Gonzalez A. Waldron L. Segata N. Knight R. Huttenhower C. Ley R. E. (2013). A guide to enterotypes across the human body: meta-analysis of microbial community structures in human microbiome datasets. PLOS Comput. Biol. 9, e1002863. 23326225 10.1371/journal.pcbi.1002863 PMC3542080

[b59-0080001] Kovacs A. Ben-Jacob N. Tayem H. Halperin E. Iraqi F. A. Gophna U. (2011). Genotype is a stronger determinant than sex of the mouse gut microbiota. Microb. Ecol. 61, 423–428. 21181142 10.1007/s00248-010-9787-2

[b60-0080001] Krych L. Hansen C. H. F. Hansen A. K. van den Berg F. W. J. Nielsen D. S. (2013). Quantitatively different, yet qualitatively alike: a meta-analysis of the mouse core gut microbiome with a view towards the human gut microbiome. PLoS ONE 8, e62578. 23658749 10.1371/journal.pone.0062578 PMC3641060

[b61-0080001] Kuehbacher T. Rehman A. Lepage P. Hellmig S. Fölsch U. R. Schreiber S. Ott S. J. (2008). Intestinal TM7 bacterial phylogenies in active inflammatory bowel disease. J. Med. Microbiol. 57, 1569–1576. 19018031 10.1099/jmm.0.47719-0

[b62-0080001] Lamendella R. Domingo J. W. S. Ghosh S. Martinson J. Oerther D. B. (2011). Comparative fecal metagenomics unveils unique functional capacity of the swine gut. BMC Microbiol. 11, 103. 21575148 10.1186/1471-2180-11-103 PMC3123192

[b63-0080001] Le Chatelier E. Nielsen T. Qin J. Prifti E. Hildebrand F. Falony G. Almeida M. Arumugam M. Batto J.-M. Kennedy S. MetaHIT consortium (2013). Richness of human gut microbiome correlates with metabolic markers. Nature 500, 541–546. 23985870 10.1038/nature12506

[b64-0080001] Lee S.-Y. Yu J. Ahn K.-M. Kim K. W. Shin Y. H. Lee K.-S. Hong S. A. Jung Y.-H. Lee E. Yang S.-I. (2014). Additive effect between IL-13 polymorphism and cesarean section delivery/prenatal antibiotics use on atopic dermatitis: a birth cohort study (COCOA). PLoS ONE 9, e96603. 24848505 10.1371/journal.pone.0096603 PMC4029558

[b65-0080001] Ley R. E. Bäckhed F. Turnbaugh P. Lozupone C. A. Knight R. D. Gordon J. I. (2005). Obesity alters gut microbial ecology. Proc. Natl. Acad. Sci. USA 102, 11070–11075. 16033867 10.1073/pnas.0504978102 PMC1176910

[b66-0080001] Ley R. E. Turnbaugh P. J. Klein S. Gordon J. I. (2006). Microbial ecology: human gut microbes associated with obesity. Nature 444, 1022–1023. 17183309 10.1038/4441022a

[b67-0080001] Li K. Bihan M. Methé B. A. (2013). Analyses of the stability and core taxonomic memberships of the human microbiome. PLoS ONE 8, e63139. 23671663 10.1371/journal.pone.0063139 PMC3646044

[b68-0080001] Linnenbrink M. Wang J. Hardouin E. A. Künzel S. Metzler D. Baines J. F. (2013). The role of biogeography in shaping diversity of the intestinal microbiota in house mice. Mol. Ecol. 22, 1904–1916. 23398547 10.1111/mec.12206

[b69-0080001] Liou A. P. Paziuk M. Luevano J.-M. Machineni S. Turnbaugh P. J. Kaplan L. M. (2013). Conserved shifts in the gut microbiota due to gastric bypass reduce host weight and adiposity. Sci. Transl. Med. 5, 178ra41–178ra41. 10.1126/scitranslmed.3005687 PMC3652229 23536013

[b70-0080001] Litten-Brown J. C. Corson A. M. Clarke L. (2010). Porcine models for the metabolic syndrome, digestive and bone disorders: a general overview. Animal 4, 899–920. 22444262 10.1017/S1751731110000200

[b71-0080001] Liu T. Hougen H. Vollmer A. C. Hiebert S. M. (2012). Gut bacteria profiles of Mus musculus at the phylum and family levels are influenced by saturation of dietary fatty acids. Anaerobe 18, 331–337. 22387300 10.1016/j.anaerobe.2012.02.004

[b72-0080001] Lupp C. Robertson M. L. Wickham M. E. Sekirov I. Champion O. L. Gaynor E. C. Finlay B. B. (2007). Host-mediated inflammation disrupts the intestinal microbiota and promotes the overgrowth of Enterobacteriaceae. Cell Host Microbe 2, 204. 18030708 10.1016/j.chom.2007.08.002

[b73-0080001] Ma Q. Lu A. Y. H. (2011). Pharmacogenetics, pharmacogenomics, and individualized medicine. Pharmacol. Rev. 63, 437–459. 21436344 10.1124/pr.110.003533

[b74-0080001] Ma A. Malynn B. A. (2012). A20: linking a complex regulator of ubiquitylation to immunity and human disease. Nat. Rev. Immunol. 12, 774–785. 23059429 10.1038/nri3313 PMC3582397

[b75-0080001] Ma B. W. Bokulich N. A. Castillo P. A. Kananurak A. Underwood M. A. Mills D. A. Bevins C. L. (2012). Routine habitat change: a source of unrecognized transient alteration of intestinal microbiota in laboratory mice. PLoS ONE 7, e47416. 23082164 10.1371/journal.pone.0047416 PMC3474821

[b76-0080001] Manichanh C. Borruel N. Casellas F. Guarner F. (2012). The gut microbiota in IBD. Nat. Rev. Gastroenterol. Hepatol. 9, 599–608. 22907164 10.1038/nrgastro.2012.152

[b77-0080001] Mariat D. Firmesse O. Levenez F. Guimarăes V. Sokol H. Doré J. Corthier G. Furet J.-P. (2009). The Firmicutes/Bacteroidetes ratio of the human microbiota changes with age. BMC Microbiol. 9, 123. 19508720 10.1186/1471-2180-9-123 PMC2702274

[b78-0080001] Martínez I. Muller C. E. Walter J. (2013). Long-term temporal analysis of the human fecal microbiota revealed a stable core of dominant bacterial species. PLoS ONE 8, e69621. 23874976 10.1371/journal.pone.0069621 PMC3712949

[b79-0080001] Maurice C. F. Haiser H. J. Turnbaugh P. J. (2013). Xenobiotics shape the physiology and gene expression of the active human gut microbiome. Cell 152, 39–50. 23332745 10.1016/j.cell.2012.10.052 PMC3552296

[b80-0080001] McCafferty J. Mühlbauer M. Gharaibeh R. Z. Arthur J. C. Perez-Chanona E. Sha W. Jobin C. Fodor A. A. (2013). Stochastic changes over time and not founder effects drive cage effects in microbial community assembly in a mouse model. ISME J. 7, 2116–2125. 23823492 10.1038/ismej.2013.106 PMC3806260

[b81-0080001] McKenna P. Hoffmann C. Minkah N. Aye P. P. Lackner A. Liu Z. Lozupone C. A. Hamady M. Knight R. Bushman F. D. (2008). The macaque gut microbiome in health, lentiviral infection, and chronic enterocolitis. PLoS Pathog. 4, e20. 18248093 10.1371/journal.ppat.0040020 PMC2222957

[b82-0080001] McNulty N. P. Yatsunenko T. Hsiao A. Faith J. J. Muegge B. D. Goodman A. L. Henrissat B. Oozeer R. Cools-Portier S. Gobert G. (2011). The impact of a consortium of fermented milk strains on the gut microbiome of gnotobiotic mice and monozygotic twins. Sci. Transl. Med. 3, 106ra106. 10.1126/scitranslmed.3002701 PMC3303609 22030749

[b83-0080001] Meng Y.-Y. Pickett M. C. Babey S. H. Davis A. C. Goldstein H. (2014). Diabetes Tied to a Third of California Hospital Stays, Driving Health Care Costs Higher, pp. 1–7, PB2014-3 Los Angeles, CA: The UCLA Center for Health Policy Research PubMed 24912203

[b84-0080001] Menon R. Watson S. E. Thomas L. N. Allred C. D. Dabney A. Azcarate-Peril M. A. Sturino J. M. (2013). Diet complexity and estrogen receptor β status affect the composition of the murine intestinal microbiota. Appl. Environ. Microbiol. 79, 5763–5773. 23872567 10.1128/AEM.01182-13 PMC3754184

[b85-0080001] Middelbos I. S. Vester Boler B. M. Qu A. White B. A. Swanson K. S. Fahey G. C. Jr (2010). Phylogenetic characterization of fecal microbial communities of dogs fed diets with or without supplemental dietary fiber using 454 pyrosequencing. PLoS ONE 5, e9768. 20339542 10.1371/journal.pone.0009768 PMC2842427

[b86-0080001] Moeller A. H. Ochman H. (2014). Microbiomes are true to type. Proc. Natl. Acad. Sci. USA 111, 9372–9373. 24938784 10.1073/pnas.1408654111 PMC4084485

[b87-0080001] Moeller A. H. Degnan P. H. Pusey A. E. Wilson M. L. Hahn B. H. Ochman H. (2012). Chimpanzees and humans harbour compositionally similar gut enterotypes. Nat. Commun. 3, 1179. 23149725 10.1038/ncomms2159 PMC3520023

[b88-0080001] Murphy E. F. Cotter P. D. Healy S. Marques T. M. O’Sullivan O. Fouhy F. Clarke S. F. O’Toole P. W. Quigley E. M. Stanton C. (2010). Composition and energy harvesting capacity of the gut microbiota: relationship to diet, obesity and time in mouse models. Gut 59, 1635–1642. 20926643 10.1136/gut.2010.215665

[b89-0080001] Nagalingam N. A. Kao J. Y. Young V. B. (2011). Microbial ecology of the murine gut associated with the development of dextran sodium sulfate-induced colitis. Inflamm. Bowel Dis. 17, 917–926. 21391286 10.1002/ibd.21462 PMC3058753

[b90-0080001] Nagy-Szakal D. Ross M. C. Dowd S. E. Mir S. A. V. Schaible T. D. Petrosino J. F. Kellermayer R. (2012). Maternal micronutrients can modify colonic mucosal microbiota maturation in murine offspring. Gut Microbes 3, 426–433. 22713270 10.4161/gmic.20697 PMC3679229

[b91-0080001] Neeb Z. P. Edwards J. M. Alloosh M. Long X. Mokelke E. A. Sturek M. (2010). Metabolic syndrome and coronary artery disease in Ossabaw compared with Yucatan swine. Comp. Med. 60, 300–315. 20819380 PMC2930329

[b92-0080001] Nell S. Suerbaum S. Josenhans C. (2010). The impact of the microbiota on the pathogenesis of IBD: lessons from mouse infection models. Nat. Rev. Microbiol. 8, 564–577. 20622892 10.1038/nrmicro2403

[b93-0080001] Nemoto H. Kataoka K. Ishikawa H. Ikata K. Arimochi H. Iwasaki T. Ohnishi Y. Kuwahara T. Yasutomo K. (2012). Reduced diversity and imbalance of fecal microbiota in patients with ulcerative colitis. Dig. Dis. Sci. 57, 2955–2964. 22623042 10.1007/s10620-012-2236-y

[b94-0080001] Neufeld K.-A. M. Kang N. Bienenstock J. Foster J. A. (2011). Effects of intestinal microbiota on anxiety-like behavior. Commun. Integr. Biol. 4, 492–494. 21966581 10.4161/cib.4.4.15702 PMC3181531

[b95-0080001] Neyrinck A. M. Possemiers S. Druart C. Van de Wiele T. De Backer F. Cani P. D. Larondelle Y. Delzenne N. M. (2011). Prebiotic effects of wheat arabinoxylan related to the increase in bifidobacteria, Roseburia and Bacteroides/Prevotella in diet-induced obese mice. PLoS ONE 6, e20944. 21695273 10.1371/journal.pone.0020944 PMC3111466

[b96-0080001] Nishihara T. Matsuda M. Araki H. Oshima K. Kihara S. Funahashi T. Shimomura I. (2006). Effect of adiponectin on murine colitis induced by dextran sulfate sodium. Gastroenterology 131, 853–861. 16952554 10.1053/j.gastro.2006.06.015

[b97-0080001] Ouellette A. J. Selsted M. E. (1996). Paneth cell defensins: endogenous peptide components of intestinal host defense. FASEB J. 10, 1280–1289. 8836041 10.1096/fasebj.10.11.8836041

[b98-0080001] Parks B. W. Nam E. Org E. Kostem E. Norheim F. Hui S. T. Pan C. Civelek M. Rau C. D. Bennett B. J. (2013). Genetic control of obesity and gut microbiota composition in response to high-fat, high-sucrose diet in mice. Cell Metab. 17, 141–152. 23312289 10.1016/j.cmet.2012.12.007 PMC3545283

[b99-0080001] Pedersen R. Ingerslev H.-C. Sturek M. Alloosh M. Cirera S. Christoffersen B. Ø. Moesgaard S. G. Larsen N. Boye M. (2013). Characterisation of gut microbiota in Ossabaw and Göttingen minipigs as models of obesity and metabolic syndrome. PLoS ONE 8, e56612. 23437186 10.1371/journal.pone.0056612 PMC3577853

[b100-0080001] Peloquin J. M. Nguyen D. D. (2013). The microbiota and inflammatory bowel disease: insights from animal models. Anaerobe 24, 102–106. 23603043 10.1016/j.anaerobe.2013.04.006 PMC3766478

[b101-0080001] Pritchett-Corning K. R. Cosentino J. Clifford C. B. (2009). Contemporary prevalence of infectious agents in laboratory mice and rats. Lab. Anim. 43, 165–173. 19015179 10.1258/la.2008.008009

[b102-0080001] Qin J. Li R. Raes J. Arumugam M. Burgdorf K. S. Manichanh C. Nielsen T. Pons N. Levenez F. Yamada T. MetaHIT Consortium (2010). A human gut microbial gene catalogue established by metagenomic sequencing. Nature 464, 59–65. 20203603 10.1038/nature08821 PMC3779803

[b103-0080001] Qin J. Li Y. Cai Z. Li S. Zhu J. Zhang F. Liang S. Zhang W. Guan Y. Shen D. (2012). A metagenome-wide association study of gut microbiota in type 2 diabetes. Nature 490, 55–60. 23023125 10.1038/nature11450

[b104-0080001] Riboulet-Bisson E. Sturme M. H. J. Jeffery I. B. O’Donnell M. M. Neville B. A. Forde B. M. Claesson M. J. Harris H. Gardiner G. E. Casey P. G. (2012). Effect of Lactobacillus salivarius bacteriocin Abp118 on the mouse and pig intestinal microbiota. PLoS ONE 7, e31113. 22363561 10.1371/journal.pone.0031113 PMC3281923

[b105-0080001] Ridaura V. K. Faith J. J. Rey F. E. Cheng J. Duncan A. E. Kau A. L. Griffin N. W. Lombard V. Henrissat B. Bain J. R. (2013). Gut microbiota from twins discordant for obesity modulate metabolism in mice. Science 341, 1241214. 24009397 10.1126/science.1241214 PMC3829625

[b106-0080001] Russell S. L. Gold M. J. Hartmann M. Willing B. P. Thorson L. Wlodarska M. Gill N. Blanchet M.-R. Mohn W. W. McNagny K. M. (2012). Early life antibiotic-driven changes in microbiota enhance susceptibility to allergic asthma. EMBO Rep. 13, 440–447. 22422004 10.1038/embor.2012.32 PMC3343350

[b107-0080001] Schreiber S. Fedorak R. N. Nielsen O. H. Wild G. Williams C. N. Nikolaus S. Jacyna M. Lashner B. A. Gangl A. Rutgeerts P. (2000). Safety and efficacy of recombinant human interleukin 10 in chronic active Crohn’s disease. Gastroenterology 119, 1461–1472. 11113067 10.1053/gast.2000.20196

[b108-0080001] Schumann S. Alpert C. Engst W. Loh G. Blaut M. (2012). Dextran sodium sulfate-induced inflammation alters the expression of proteins by intestinal Escherichia coli strains in a gnotobiotic mouse model. Appl. Environ. Microbiol. 78, 1513–1522. 22210207 10.1128/AEM.07340-11 PMC3294480

[b109-0080001] Schwiertz A. Taras D. Schäfer K. Beijer S. Bos N. A. Donus C. Hardt P. D. (2010). Microbiota and SCFA in lean and overweight healthy subjects. Obesity (Silver Spring) 18, 190–195. 19498350 10.1038/oby.2009.167

[b110-0080001] Serino M. Luche E. Gres S. Baylac A. Bergé M. Cenac C. Waget A. Klopp P. Iacovoni J. Klopp C. (2012). Metabolic adaptation to a high-fat diet is associated with a change in the gut microbiota. Gut 61, 543–553. 22110050 10.1136/gutjnl-2011-301012 PMC3292714

[b111-0080001] Smith M. I. Yatsunenko T. Manary M. J. Trehan I. Mkakosya R. Cheng J. Kau A. L. Rich S. S. Concannon P. Mychaleckyj J. C. (2013a). Gut microbiomes of Malawian twin pairs discordant for kwashiorkor. Science 339, 548–554. 23363771 10.1126/science.1229000 PMC3667500

[b112-0080001] Smith H. F. Parker W. Kotzé S. H. Laurin M. (2013b). Multiple independent appearances of the cecal appendix in mammalian evolution and an investigation of related ecological and anatomical factors. C. R. Palevol 12, 339–354.

[b113-0080001] Soave O. Brand C. D. (1991). Coprophagy in animals: a review. Cornell Vet. 81, 357–364. 1954740

[b114-0080001] Sokol H. Seksik P. Rigottier-Gois L. Lay C. Lepage P. Podglajen I. Marteau P. Doré J. (2006). Specificities of the fecal microbiota in inflammatory bowel disease. Inflamm. Bowel Dis. 12, 106–111. 16432374 10.1097/01.MIB.0000200323.38139.c6

[b115-0080001] Sokol H. Pigneur B. Watterlot L. Lakhdari O. Bermúdez-Humarán L. G. Gratadoux J.-J. Blugeon S. Bridonneau C. Furet J.-P. Corthier G. (2008). Faecalibacterium prausnitzii is an anti-inflammatory commensal bacterium identified by gut microbiota analysis of Crohn disease patients. Proc. Natl. Acad. Sci. USA 105, 16731–16736. 18936492 10.1073/pnas.0804812105 PMC2575488

[b116-0080001] Sokol H. Seksik P. Furet J. P. Firmesse O. Nion-Larmurier I. Beaugerie L. Cosnes J. Corthier G. Marteau P. Doré J. (2009). Low counts of Faecalibacterium prausnitzii in colitis microbiota. Inflamm. Bowel Dis. 15, 1183–1189. 19235886 10.1002/ibd.20903

[b117-0080001] Spor A. Koren O. Ley R. (2011). Unravelling the effects of the environment and host genotype on the gut microbiome. Nat. Rev. Microbiol. 9, 279–290. 21407244 10.1038/nrmicro2540

[b118-0080001] Suchodolski J. S. Camacho J. Steiner J. M. (2008). Analysis of bacterial diversity in the canine duodenum, jejunum, ileum, and colon by comparative 16S rRNA gene analysis. FEMS Microbiol. Ecol. 66, 567–578. 18557939 10.1111/j.1574-6941.2008.00521.x

[b119-0080001] Suchodolski J. S. Dowd S. E. Westermarck E. Steiner J. M. Wolcott R. D. Spillmann T. Harmoinen J. A. (2009). The effect of the macrolide antibiotic tylosin on microbial diversity in the canine small intestine as demonstrated by massive parallel 16S rRNA gene sequencing. BMC Microbiol. 9, 210. 19799792 10.1186/1471-2180-9-210 PMC2759960

[b120-0080001] Swanson K. S. Dowd S. E. Suchodolski J. S. Middelbos I. S. Vester B. M. Barry K. A. Nelson K. E. Torralba M. Henrissat B. Coutinho P. M. (2011). Phylogenetic and gene-centric metagenomics of the canine intestinal microbiome reveals similarities with humans and mice. ISME J. 5, 639–649. 20962874 10.1038/ismej.2010.162 PMC3105739

[b121-0080001] Tap J. Mondot S. Levenez F. Pelletier E. Caron C. Furet J. P. Ugarte E. Muñoz-Tamayo R. Paslier D. L. Nalin R. (2009). Towards the human intestinal microbiota phylogenetic core. Environ. Microbiol. 11, 2574–2584. 19601958 10.1111/j.1462-2920.2009.01982.x

[b122-0080001] Taylor N. S. Xu S. Nambiar P. Dewhirst F. E. Fox J. G. (2007). Enterohepatic Helicobacter species are prevalent in mice from commercial and academic institutions in Asia, Europe, and North America. J. Clin. Microbiol. 45, 2166–2172. 17507523 10.1128/JCM.00137-07 PMC1933014

[b123-0080001] Treuting P. M. Dintzis S. M. (2012). Lower Gastrointestinal Tract, In Comparative Anatomy and Histology – a Mouse and Human Atlas, 1st edn (ed. Dintzis S. M. Frevert C. W. Liggitt H. D. Montine K. S. Treuting P. M.), Chapter 12 Amsterdam: Elsevier Inc.

[b124-0080001] Turnbaugh P. J. Ley R. E. Mahowald M. A. Magrini V. Mardis E. R. Gordon J. I. (2006). An obesity-associated gut microbiome with increased capacity for energy harvest. Nature 444, 1027–1031. 17183312 10.1038/nature05414

[b125-0080001] Turnbaugh P. J. Hamady M. Yatsunenko T. Cantarel B. L. Duncan A. Ley R. E. Sogin M. L. Jones W. J. Roe B. A. Affourtit J. P. (2009a). A core gut microbiome in obese and lean twins. Nature 457, 480–484. 19043404 10.1038/nature07540 PMC2677729

[b126-0080001] Turnbaugh P. J. Ridaura V. K. Faith J. J. Rey F. E. Knight R. Gordon J. I. (2009b). The effect of diet on the human gut microbiome: a metagenomic analysis in humanized gnotobiotic mice. Sci. Transl. Med. 1, 6ra14. 10.1126/scitranslmed.3000322 PMC2894525 20368178

[b127-0080001] Ubeda C. Bucci V. Caballero S. Djukovic A. Toussaint N. C. Equinda M. Lipuma L. Ling L. Gobourne A. No D. (2013). Intestinal microbiota containing Barnesiella species cures vancomycin-resistant Enterococcus faecium colonization. Infect. Immun. 81, 965–973. 23319552 10.1128/IAI.01197-12 PMC3584866

[b128-0080001] Uhlig H. H. Powrie F. (2009). Mouse models of intestinal inflammation as tools to understand the pathogenesis of inflammatory bowel disease. Eur. J. Immunol. 39, 2021–2026. 19672896 10.1002/eji.200939602

[b129-0080001] Vaahtovuo J. Munukka E. Korkeamäki M. Luukkainen R. Toivanen P. (2008). Fecal microbiota in early rheumatoid arthritis. J. Rheumatol. 35, 1500–1505. 18528968

[b130-0080001] Velcich A. Yang W. Heyer J. Fragale A. Nicholas C. Viani S. Kucherlapati R. Lipkin M. Yang K. Augenlicht L. (2002). Colorectal cancer in mice genetically deficient in the mucin Muc2. Science 295, 1726–1729. 11872843 10.1126/science.1069094

[b131-0080001] Vereecke L. Vieira-Silva S. Billiet T. van Es J. H. McGuire C. Slowicka K. Sze M. van den Born M. De Hertogh G. Clevers H. (2014). A20 controls intestinal homeostasis through cell-specific activities. Nat. Commun. 5, 5103. 25267258 10.1038/ncomms6103

[b132-0080001] Vigsnæs L. K. Brynskov J. Steenholdt C. Wilcks A. Licht T. R. (2012). Gram-negative bacteria account for main differences between faecal microbiota from patients with ulcerative colitis and healthy controls. Benef. Microbes 3, 287–297. 22968374 10.3920/BM2012.0018

[b133-0080001] Walker A. W. Sanderson J. D. Churcher C. Parkes G. C. Hudspith B. N. Rayment N. Brostoff J. Parkhill J. Dougan G. Petrovska L. (2011). High-throughput clone library analysis of the mucosa-associated microbiota reveals dysbiosis and differences between inflamed and non-inflamed regions of the intestine in inflammatory bowel disease. BMC Microbiol. 11, 7. 21219646 10.1186/1471-2180-11-7 PMC3032643

[b134-0080001] Wang L. Christophersen C. T. Sorich M. J. Gerber J. P. Angley M. T. Conlon M. A. (2013). Increased abundance of Sutterella spp. and Ruminococcus torques in feces of children with autism spectrum disorder. Mol. Autism 4, 42. 24188502 10.1186/2040-2392-4-42 PMC3828002

[b135-0080001] Wang J. Linnenbrink M. Künzel S. Fernandes R. Nadeau M.-J. Rosenstiel P. Baines J. F. (2014). Dietary history contributes to enterotype-like clustering and functional metagenomic content in the intestinal microbiome of wild mice. Proc. Natl. Acad. Sci. USA 111, E2703–E2710. 24912178 10.1073/pnas.1402342111 PMC4084472

[b136-0080001] Ward N. L. Pieretti A. Dowd S. E. Cox S. B. Goldstein A. M. (2012). Intestinal aganglionosis is associated with early and sustained disruption of the colonic microbiome. Neurogastroenterol. Motil. 24, 874–e400. 22626027 10.1111/j.1365-2982.2012.01937.x

[b137-0080001] Werner T. Wagner S. J. Martínez I. Walter J. Chang J.-S. Clavel T. Kisling S. Schuemann K. Haller D. (2011). Depletion of luminal iron alters the gut microbiota and prevents Crohn’s disease-like ileitis. Gut 60, 325–333. 21076126 10.1136/gut.2010.216929

[b138-0080001] Wirtz S. Neurath M. F. (2007). Mouse models of inflammatory bowel disease. Adv. Drug Deliv. Rev. 59, 1073–1083. 17825455 10.1016/j.addr.2007.07.003

[b139-0080001] Willing B. Halfvarson J. Dicksved J. Rosenquist M. Järnerot G. Engstrand L. Tysk C. Jansson J. K. (2009). Twin studies reveal specific imbalances in the mucosa-associated microbiota of patients with ileal Crohn’s disease. Inflamm. Bowel Dis. 15, 653–660. 19023901 10.1002/ibd.20783

[b140-0080001] Willing B. P. Dicksved J. Halfvarson J. Andersson A. F. Lucio M. Zheng Z. Järnerot G. Tysk C. Jansson J. K. Engstrand L. (2010). A pyrosequencing study in twins shows that gastrointestinal microbial profiles vary with inflammatory bowel disease phenotypes. Gastroenterology 139, 1844–1854.e1. 20816835 10.1053/j.gastro.2010.08.049

[b141-0080001] Wirtz S. Neurath M. F. (2007). Mouse models of inflammatory bowel disease. Adv. Drug Deliv. Rev. 59, 1073–1083. 17825455 10.1016/j.addr.2007.07.003

[b142-0080001] Wirtz S. Neufert C. Weigmann B. Neurath M. F. (2007). Chemically induced mouse models of intestinal inflammation. Nat. Protoc. 2, 541–546. 17406617 10.1038/nprot.2007.41

[b143-0080001] Wohlgemuth S. Haller D. Blaut M. Loh G. (2009). Reduced microbial diversity and high numbers of one single Escherichia coli strain in the intestine of colitic mice. Environ. Microbiol. 11, 1562–1571. 19245530 10.1111/j.1462-2920.2009.01883.x

[b144-0080001] Wos-Oxley M. Bleich A. Oxley A. P. A. Kahl S. Janus L. M. Smoczek A. Nahrstedt H. Pils M. C. Taudien S. Platzer M. (2012). Comparative evaluation of establishing a human gut microbial community within rodent models. Gut Microbes 3, 234–249. 22572831 10.4161/gmic.19934 PMC3427216

[b145-0080001] Wu G. D. Chen J. Hoffmann C. Bittinger K. Chen Y. Y. Keilbaugh S. A. Bewtra M. Knights D. Walters W. A. Knight R. (2011). Linking long-term dietary patterns with gut microbial enterotypes. Science 334, 105–108. 21885731 10.1126/science.1208344 PMC3368382

[b146-0080001] Würbel H. (2000). Behaviour and the standardization fallacy. Nat. Genet. 26, 263. 11062457 10.1038/81541

[b147-0080001] Yanabe M. Shibuya M. Gonda T. Asai H. Tanaka T. Sudou K. Narita T. Matsui T. Itoh K. (2001). Establishment of specific pathogen-free guinea-pig colonies using limited-flora guinea-pigs associated with conventional guinea-pig flora, and monitoring of their cecal flora. Exp. Anim. 50, 105–113. 11381613 10.1538/expanim.50.105

[b148-0080001] Yang Z. Zhang N. (2014). The burden of overweight and obesity on long-term care and Medicaid financing. Med. Care 52, 658–663. 24926714 10.1097/MLR.0000000000000154

[b149-0080001] Yatsunenko T. Rey F. E. Manary M. J. Trehan I. Dominguez-Bello M. G. Contreras M. Magris M. Hidalgo G. Baldassano R. N. Anokhin A. P. (2012). Human gut microbiome viewed across age and geography. Nature 486, 222–227. 22699611 10.1038/nature11053 PMC3376388

[b150-0080001] Zenewicz L. A. Yin X. Wang G. Elinav E. Hao L. Zhao L. Flavell R. A. (2013). IL-22 deficiency alters colonic microbiota to be transmissible and colitogenic. J. Immunol. 190, 5306–5312. 23585682 10.4049/jimmunol.1300016 PMC3646987

[b151-0080001] Zhang C. Zhang M. Pang X. Zhao Y. Wang L. Zhao L. (2012). Structural resilience of the gut microbiota in adult mice under high-fat dietary perturbations. ISME J. 6, 1848–1857. 22495068 10.1038/ismej.2012.27 PMC3446802

