# Supplementary material for: How informative is the mouse for human gut microbiota research?
Source: Dis Model Mech. 2015 Jan;8(1):1–16. doi: 10.1242/dmm.017400 (PMC4283646; doi:10.1242/dmm.017400)
Supplement: Supplementary Material [file supp_8_1_1__index.html]

How informative is the mouse for human gut microbiota research? — Supplementary Material 

# How informative is the mouse for human gut microbiota research?

## DMM017400 Supplementary Material

**Files in this Data Supplement:**

- **Supplementary Material**
