# Supplementary material for: How informative is the mouse for human gut microbiota research?
Source: Dis Model Mech. 2015 Jan;8(1):1–16. doi: 10.1242/dmm.017400 (PMC4283646; doi:10.1242/dmm.017400)
Supplement: Supplementary Material [file supp_8.1.1_DMM017400.pdf]

Tables

Supplementary Table 1: Genera in common between mice and humans gut microbiota. Top 10 most abundant genera present in at least 1 of the 4 human studies (Consortium, 2012; Yatsunenko et al., 2012; left column) and 1 of the 5 mouse gut microbiota datasets (Nagy-Szakal et al., 2012; Riboulet-Bisson et al., 2012; Ubeda et al., 2013; Ward et al., 2012; Zenewicz et al., 2013; right column) that were used for the analysis reported in Figure 3. Genera with significantly different abundances between human and mouse datasets are indicated with an asterisk (Wilcoxon p-value <0.05), but note that none are significant after multiple testing correction. Low-abundance genera with significantly different abundances between human and mouse datasets are listed below. For each dataset, genus abundances were normalized to the most abundant genus (100%). The mean relative abundance of each genus was calculated as its average relative abundance in all the datasets in which the genus was detected.

**Mouse TOP 10**			**Human TOP 10**		
**Genus**	**Mean relative abundance (%)**		**Genus**	**Mean relative abundance (%)**	
	**mice**	**humans**		**mice**	**humans**
*Lactobacillus**	63.136	0.047	*Prevotella*	0.869	51.672
Clostridium	32.243	22.072	*Faecalibacterium**	0.271	49.409
Bacteroides	25.412	37.475	*Bacteroides*	25.412	37.475
Alistipes	25.241	7.554	*Ruminococcus**	1.977	26.270
*Turicibacter**	22.553	0.222	*Clostridium*	32.243	22.072
Blautia	15.047	20.266	*Blautia*	15.047	20.266
Eubacterium	8.944	9.472	*Roseburia*	3.915	18.156
Parabacteroides	7.478	5.249	*Coprococcus*	1.931	11.972
Tannerella	5.320	0.001	*Eubacterium*	8.944	9.472
Roseburia	3.915	18.156	*Oscillospira**	1.075	7.708
**Low-abundance genera with significant abundance difference between humans and mice**					
	**mice**	**humans**		**mice**	**humans**
*Catenibacterium**	0.031	0.874	*Akkermansia**	0.003	0.744
*Lachnospira**	0.230	3.502	*Haemophilus**	0.002	0.028
*Streptococcus**	0.066	0.962	*Klebsiella**	0.002	0.840
*Catenibacterium**	0.031	0.874	*Dialister**	0.002	3.856

Supplementary Table 2: Alphabetical list of mouse- and human-specific gut-associated genera. Data was extracted from the same four human studies used for Figure 3 and Supplementary Table 1 (Consortium, 2012; Yatsunenko et al., 2012) and six mouse studies used for Figure 3B (Cho et al., 2012; Nagy-Szakal et al., 2012; Riboulet-Bisson et al., 2012; Ubeda et al., 2013; Ward et al., 2012; Zenewicz et al., 2013). Genera with less than 0.1% relative abundance were excluded. Genera with significantly different abundances between the human and mouse datasets (referenced in Supplementary Table 1) are indicated with an asterisk (Wilcoxon p-value <0.05), but note that none is significant after multiple testing correction.

Mouse-specific			Human-specific
**Genera**	**Genera (cont.)**	**Genera (cont.)**	**Genera**
Acetanaerobacterium	*Croceibacter*	*Oscillibacter*	*Acidaminococcus*
Acetitomaculum	*Curvibacter*	*Paludibacter*	*Adlercreutzia**
Aeromicrobium	*Cytophaga*	*Papillibacter*	*Bilophila**
Alkaliphilus	*Cytophagaceae*	*Parasporobacterium*	*Bulleidia**
*Allobaculum**	*Desulfonispora*	*Pelagibacter*	*Collinsella**
Anaerobacter	*Donghicola*	*Petrimonas*	*Erwinia**
Anaerophaga	*Erysipelothrix*	*Rhodovulum*	*Methanobrevibacter**
Anaeroplasma	*Eubacteriaceae*	*Rikenella*	*Odoribacter**
Anaerosporobacter	*Guggenheimella*	*Roseobacter*	*Phascolarctobacterium*
*Anaerovorax**	*Hespellia*	*Ruminococcaceae I.S*	*Sutterella**
Arthromitus	*Johnsonella*	*Shigella*	*Veillonella**
Bryantella	*Lachnospiraceae*	*Sporacetigenium*	
Butyricicoccus	*Lachnospiraceae (IS)*	*Sporobacter*	
Caminicella	*Leifsonia*	*Sporobacterium*	
Catonella	*Longispora*	*Syntrophococcus*	
Clostridiaceae	*Marvinbryantia*	*Tessaracoccus*	
Clostridiales	*Mucispirillum*	*TM7 genera I.S*	
